# Histopathological evaluation of a retinoic acid eluting stent in a rabbit iliac artery model

**DOI:** 10.1038/s41598-022-16025-5

**Published:** 2022-08-03

**Authors:** Ioanna Samara, Christos S. Katsouras, Arsen Semertzioglou, Athanassios Vratimos, Amalia I. Moula, Constantinos A. Dimitriou, Michail Theofanis, Triantafyllia Papadimitropoulou, Vasileios Bouratzis, Georgia Karanasiou, Dimitrios Fotiadis, Lampros K. Michalis, Anargyros N. Moulas

**Affiliations:** 1 2nd Department of Cardiology, University Hospital of Ioannina, Ioannina, Greece; 2 Faculty of Medicine, School of Health Sciences, University of Ioannina, Ioannina, Greece; 3 Rontis Corporation, Zug, Switzerland; 4 General Department, University of Thessaly, Larissa, Greece; 5 Faculty of Health Medicine and Life Sciences, University of Maastricht, Maastricht, The Netherlands; 6 Center for Clinical, Experimental Surgery and Translational Research, Biomedical Research Foundation of the Academy of Athens, Athens, Greece; 7 Department of Interventional Radiology, University Hospital of Patras, Patras, Greece; 8 Department of Biomedical Research, Institute of Molecular Biology and Biotechnology, University of Ioannina, Ioannina, Greece; 9 Unit of Medical Technology and Intelligent Information Systems, Department of Materials Science and Engineering, University of Ioannina, Ioannina, Greece

**Keywords:** Cardiac device therapy, Interventional cardiology, Experimental models of disease, Preclinical research

## Abstract

This study aimed to evaluate the safety and efficacy of innovative retinoic acid (RA) eluting stents with bioabsorbable polymer. Sixty stents divided in ten groups were implanted in the iliac arteries of 30 rabbits. Two polymers (“A”, poly (lactic-co-glycolic acid) and “B”, polylactic acid), and three doses (“Low”, “Medium” and “High”) of RA (groups: AL, AM, AH, BL, BM, BH) were used on cobalt chromium stents (Rontis Corporation), one group of bare stent (C), one group (D) of Everolimus eluting stent (Xience-Pro, Abbot Vascular), and two groups of Rontis Everolimus eluting stents coated with polymer A (EA) and B (EB). Treated arteries were explanted after 4 weeks, processed by methyl methacrylate resin and evaluated by histopathology. None of the implanted stents was related with thrombus formation or extensive inflammation. Image analysis showed limited differences between groups regarding area stenosis (BH, D and EB groups had the lower values). Group BH had lower intimal mean thickness than AH (105.1 vs 75.3 μm, p = 0.024). Stents eluting RA, a non-cytotoxic drug, were not related with thrombus formation and had an acceptable degree of stenosis 4 weeks post implantation. RA dose and type of polymer may play role in the biocompatibility of the stents.

## Introduction

Implantation of new-generation drug eluting stent (DES) is the standard treatment strategy in percutaneous coronary intervention^1^. The devices release an antiproliferative drug locally, thereby acting against the proliferation of smooth muscle cells, directly in the endothelium preventing restenosis. Currently used drugs on DES include paclitaxel, a cytotoxic drug and rapamycin (sirolimus) and its derivatives such as everolimus, a group of cytostatic drugs^2^. Although the wide use of DES significantly reduced restenosis compared with bare metal stents (BMS), restenosis still is a major complication^3^. Moreover, the late and very late stent thrombosis, the need for dual antiplatelet therapy and the chronic inflammatory response to polymer and/or the cytotoxic and cytostatic drugs that are currently in use, justify further investigation and development of technologies which will improve the safety and efficacy of DES^4^.

Retinoid acid (RA) belongs to the group of retinoids, which are derivatives of vitamin A^5^. RA is a product of oxidation of retinol or retinal. The main circulating form of RA in humans is all-trans-retinoic acid, while 13-cis- and 9-cis-isomers are also present in small concentrations. RA constitutes the drastic form of vitamin A that acts through specific nuclear receptors [retinoic acid receptors (RAR) and retinoic X receptors (RXR)], affecting the expression of specific genes^5^. Retinoids contribute to the homeostasis of cell differentiation and proliferation and have been used successfully in the treatment of diseases related to cell proliferation as well as in the treatment of various malignancies, such as acute promyelocytic leukemia^6^. Studies have shown that RA has a regulatory effect on the maturation and differentiation of vascular cells, inhibition of cell proliferation, migration, inflammation and extracellular remodeling synthesis of vascular smooth muscle cells (VSMCs)^7^.

Oral administration of RA resulted in reduction of vascular stenosis in experimental in vivo stenosis models^8,9^. Moreover, local application of RA in blood vessels reduced neointimal hyperplasia in experimental models of restenosis^10,11^. The doses however that are needed to obtain these effects may cause serious side effects^12^. We hypothesized that local delivery of relatively low doses of retinoic acid using stents as the delivery platform/device, may result in reduction of restenosis after stent implantation, potentially without causing the inflammatory effects that are common with stents coated with cytostatic or cytotoxic drugs such as rapamycin and its derivatives and paclitaxel. In order to test this hypothesis, we developed innovative stents coated with bioabsorbable polymers and all-trans-retinoic acid. The aim of this study was the evaluation of the safety and efficacy of stents that release retinoid acid, in a rabbit iliac artery model.

## Results

**Figure 1:**
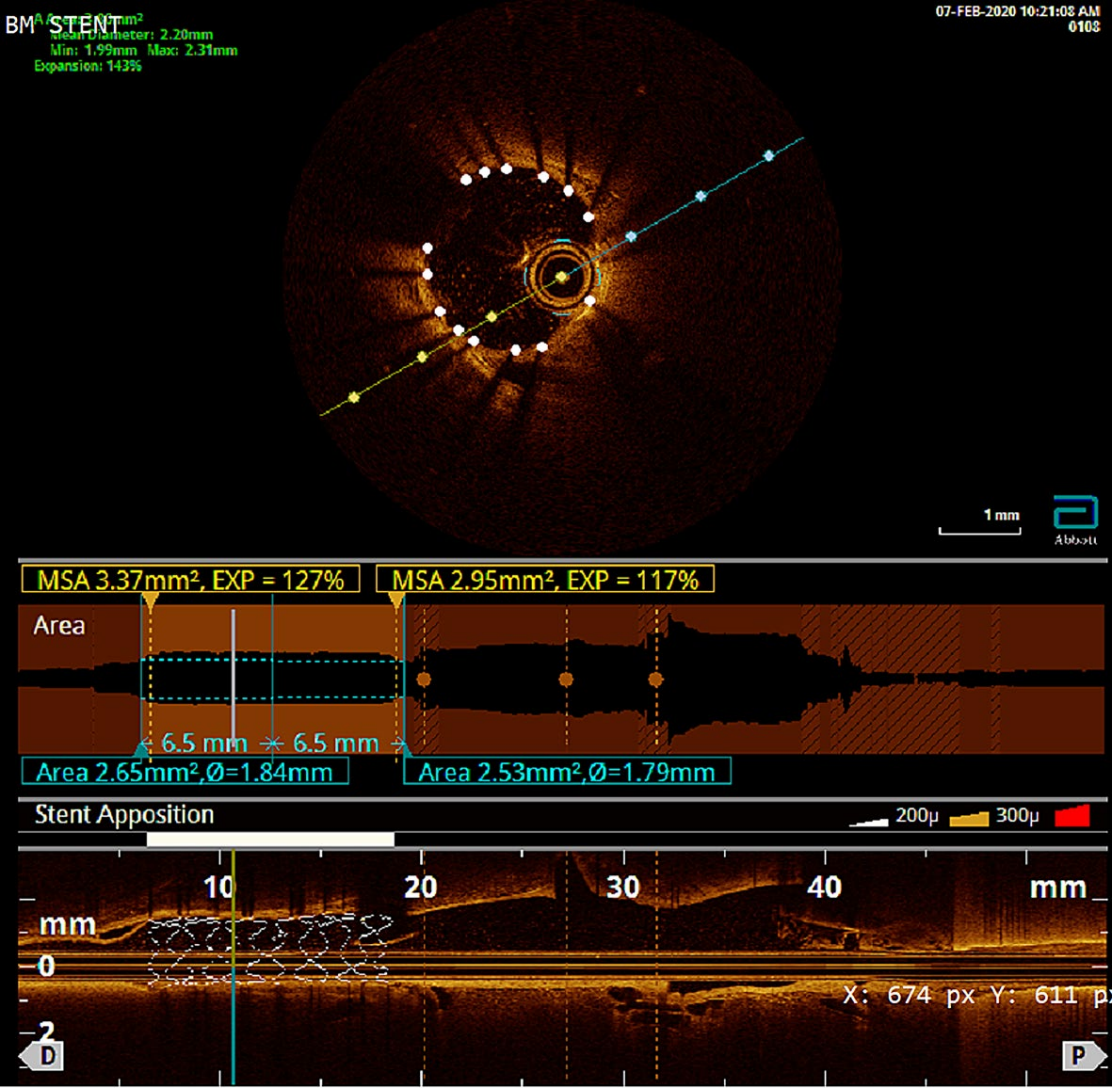
Typical optical coherence tomography (OCT) images obtained immediately after implantation of a stent of the group BM (stent with PLA polymer and retinoic acid drug).

All the stents were successfully expanded into the target arteries and the apposition was confirmed in all cases by angiography. The apposition was also evaluated by OCT after implantation in four stents. A typical OCT image of a RA coated stent of the BM group is presented in Fig. 1.

One of the animals that had received two bare metal stents did not survive until the 4-week study period and therefore was not evaluated in this study. There were no gross lesions noted at the necropsy.

None of the implanted stents was related with thrombus formation or necrosis of the vascular wall. Further, the arterial wall did not appear dissected, and only multifocal ruptures of the elastic laminae (mostly the internal one) were observed. The host reaction was considered low with all the treatments. There were unstained particles detected within the tunica intima in association with macrophages and giant cells in all the groups, except for group C (BMSs).

Markers of local inflammation were included in the histological analysis of target/stented arterial segments. All implant sites showed a low-level arterial reaction consisting of one or more of the following parameters, summarized in Table 1. The lining of the vascular wall included multifocal regions rich in reactive hypertrophic endothelial cells. The intima was thickened, resulting in local stenosis, due to the migration and proliferation of smooth muscle cells (inflammation is, possibly, a contributing factor for this expected vascular reaction). The presence of inflammation in all three layers of the vascular wall (intima, media, adventitia) was also demonstrated through the scattered presence of mixed cell infiltrates, comprised mostly of mononuclear cells. Host reaction to the implant was evident through the presence of mixed cell infiltrates, comprised of lymphocytes, polymorphonuclears, macrophages and, on occasion, of giant cells, associated with stent struts with various degrees of severity. A few implant sites presented with fibrosis, new vessel formation (neovascularization) and local haemorrage.

**Table 1:** Summarized average scores for all evaluated parameters and the total arterial reaction.

	Group									
	AL	AM	AH	BL	BM	BH	C	D	EF	EG
Vascular wall findings	10.7	9.7	12.7	10.0	7.5	9.7	10.3	7.7	8.0	8.7
Artery inflammation	2.0	2.0	2.7	2.3	3.0	2.3	2.3	2.3	3.3	2.0
Medial smooth muscle cell (SMC) loss	0.0	0.0	0.0	0.0	0.0	0.3	0.0	0.3	0.3	0.0
Medial smooth muscle cell replacement tissue	0.0	0.0	0.0	0.0	0.0	0.3	0.0	0.3	0.3	0.0
Medial hypertrophy	0.0	0.0	0.0	0.0	0.0	0.0	0.0	0.0	0.0	0.0
Lamina elastic rupture	2.3	2.3	1.7	2.3	2.0	2.3	2.3	2.0	2.3	2.0
Host reaction associated with the implant	7.7	8.5	6.8	6.7	6.5	7.7	9.2	8.8	8.3	7.8
Total arterial reaction scoring	22.7	22.5	23.8	21.3	19.0	22.3	24.2	21.2	22.3	20.5
Stainless particles	0.7	1.2	1.0	1.5	1.3	1.7	0.0	0.5	0.2	1.0

*A* polymer A, *PLGA* poly (lactic-*co*-glycolic acid), *B* polymer B, *PLA* poly lactic acid, *L* low (dose RA), *M* medium (dose RA), *H* high (dose RA), *C* Rontis bare metal stent, *D* Xience Pro everolimus eluting coronary stent system, Abbot vascular, *EF and EG* Rontis everolimus eluting stents with the use of polymers A and B respectively.

The summarized average scores for all evaluated parameters and the total arterial reaction are summarized in Table 1. Regarding the total arterial reaction score, Group BM showed the lowest values (19.0), while group C showed the highest one (24.2). The difference between these two groups was + 5.2, which was considered low. The decrease in total arterial average score in group BM compared to Group C was related to a decrease severity of the intimal proliferation, less severe ruptures of the elastic laminae and lower host reaction associated with the stent struts.

The total arterial reaction average score was lower with treatments BL, BM and BH, compared to groups AL, AM, AH. The decrease in average score was mostly due the less severe vascular wall findings and the lack or lower severity grade of peri-strut hemorrhages in BL, BM and BH groups. EF and EG groups showed less total arterial reaction score than C group. Group EF showed slightly higher average score than group EG.

**Figure 2:**
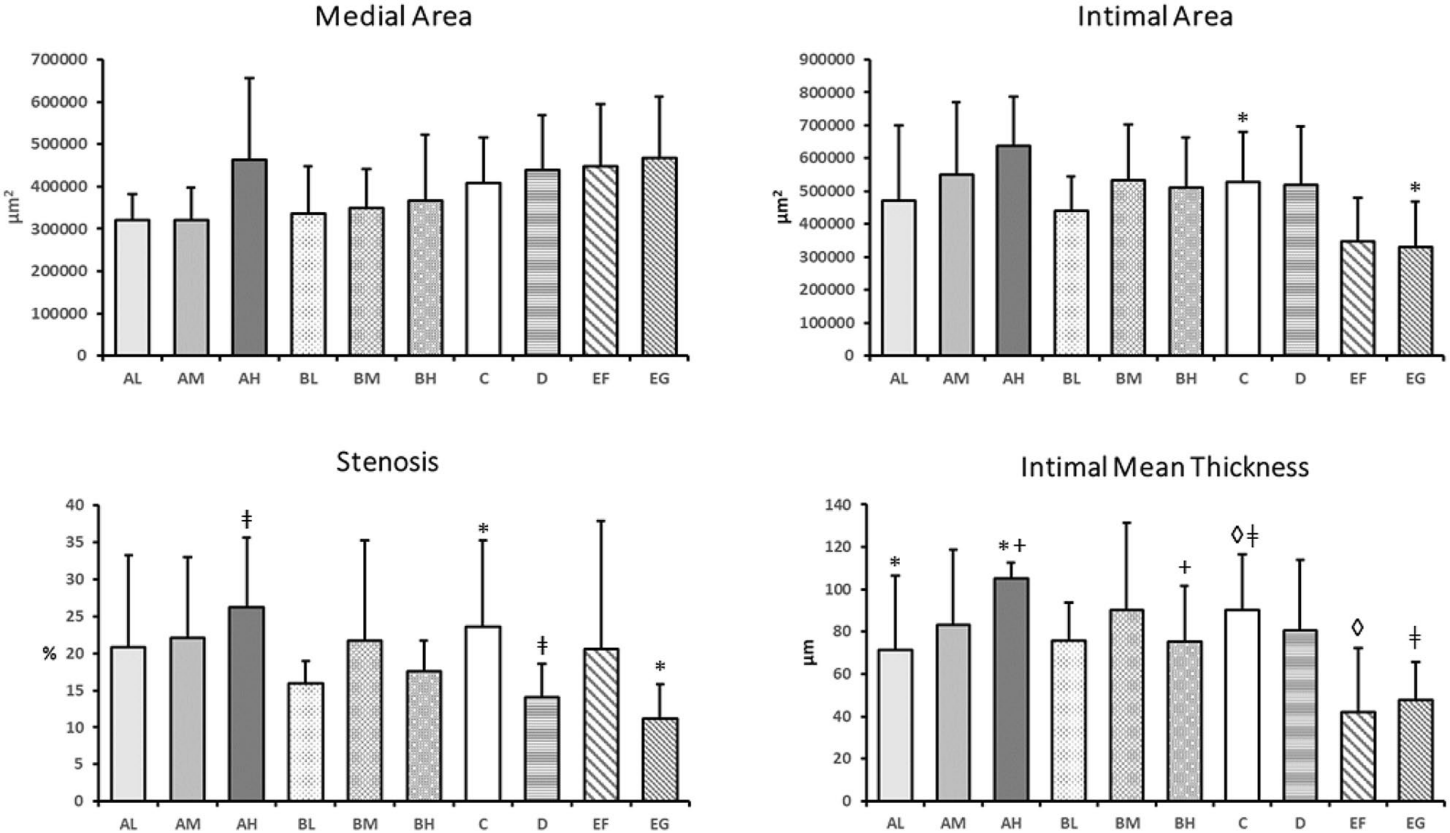
Column bar graph of combined group arithmetic mean for medial area, intimal area, stenosis, and intimal mean thickness measurements. Groups with the same symbol (asterisk, plus, diamond, double dagger) differ significantly (asterisk, plus : p < 0.05, diamond, double dagger : p < 0.02). *A* Polymer A; *PLGA* poly (lactic-*co*-glycolic acid), *B* polymer B, *PLA* poly lactic acid, *L* low (dose); *M* medium (dose), *H* high (dose); *C* Rontis bare metal stent; *D* Xience Pro everolimus eluting coronary stent system, Abbot vascular; *EF and EG* Rontis everolimus stents with the use of polymers A and B respectively. *RA* retinoic acid.

The results of image analyses are summarized in Table 2 and Fig. 2. No statistically significant difference between Groups AL, AM, AH, BL, BM and BH against Group C was present for Medial Area, Intimal Area, Stenosis and Intimal Mean Thickness. No statistically significant difference between Groups AL, AM, BL, BM and BH against Group D was present for Medial Area, Intimal Area, Stenosis and Intimal Mean Thickness.

**Table 2:** Total Summarized measurements for each group.

	AL	AM	AH	BL	BM	BH	C	D	EF	EG
**Arithmetic mean**										
Area within EEL (µm^2^)	2,736,540	3,003,017	3,103,040	3,113,391	3,185,635	3,222,887	3,024,821	4,174,413	2,719,017	3,394,371
Area within IEL (µm^2^)	2,414,954	2,681,431	2,640,787	2,776,284	2,836,129	2,856,765	2,616,252	3,735,426	2,270,365	2,926,605
Lumen area (µm^2^)	1,944,143	2,132,240	2,003,479	2,336,045	2,302,719	2,347,036	2,088,513	3,215,022	1,924,209	2,596,651
Medial area (µm^2^)	321,585	321,586	462,253	337,107	349,506	366,122	408,570	438,986	448,652	467,766
Intimal area (µm^2^)	470,811	549,191	637,308	440,239	533,410	509,729	527,738	520,404	346,156	329,954
Stenosis (%)	20.87	22.02	26.22	15.92	21.76	17.63	23.59	14.04	20.62	11.23
Intimal mean thickness (µm)	71.46	83.26	105.10	75.69	90.38	75.34	90.25	80.71	42.17	47.60

*EEL* external elastic lamina, *IEL* internal elastic lamina, *A* polymer A, *PLGA* poly (lactic-*co*-glycolic acid), *B* polymer B, *PLA* poly lactic acid, *L* low (dose RA), *M* medium (dose RA), *H* high (dose RA), *C* Rontis bare metal stent, *D* Xience Pro everolimus eluting coronary stent system, Abbot vascular, *EF and EG* Rontis everolimus eluting stents with the use of polymers A and B respectively.

Group AH showed statistically significant difference with Group BH for Intimal Mean Thickness (105.1 vs 75.3 μm^2^ respectively, p = 0.0235). Moreover, Group AH showed the highest Stenosis (26.2%) and Intimal Mean Thickness values (105.1 μm^2^). Also, Group AH showed statistically significant difference with Group D for stenosis (26.2% vs 14.0%, p = 0.0169).

Group AL showed statistically significant difference with Group AH for intimal mean thickness (71.5 vs 105.1 μm^2^ respectively, p = 0.0432). No statistically significant difference was observed between Groups BL and BH for all parameters measured.

No statistically significant difference between Groups EF and EG was present for Medial Area, Intimal Area, Stenosis and Intimal Mean Thickness. No statically significant difference was observed between Groups EF and EG and Group D, for none of the tested parameters. However, a statistically significant difference was observed between Groups EG and C for Intimal Area (329,954 vs 527,738 μm^2^, p = 0.0407) and Stenosis (11.2% vs 23.6%, p = 0.0366) and between Groups EG and C (47.6 vs 90.3 μm, p = 0.0085) and Groups EF and C for Intimal Mean Thickness (42.2 vs 90.3 μm, p = 0.0149).

## Discussion

In our experimental study, when three different doses of the drug RA and two polymers were used in stents implanted in rabbit iliac arteries, the main histopathological observations were: (1) none of the implanted stents was related with thrombus formation or necrosis of the vascular wall; therefore, 4 weeks post implantation, none of the treatments seem to increase the risk of a thrombotic event; (2) all the groups presented multifocal ruptures of the elastic laminae (mostly the internal one), but without further medial/transmural rupture, which suggested a low risk of arterial transmural dissection induced by the stent implantation at this time-point; (3) the host reaction associated with the implant struts was considered low with all the treatments with only minimal to slight differences among them; and (4) the dose of RA and the type of the polymer may play a role in the biocompatibility of the device.

Retinoids exhibit complex effects on vascular smooth muscle cells including regulation of proliferation, growth, differentiation and migration, as shown by in vitro and in vivo experiments^13^. All-trans-retinoic acid induces a dose-dependent inhibitory effect on the proliferation of human arterial smooth muscle cells *in vitro*^14,15^. A similar dose-dependent inhibition of the growth activity was also observed in human vein smooth muscle cells^9^.

Experiments with various in vivo stenosis models have shown that oral administration of retinoic acid resulted in reduction of stenosis. Oral administration of RA (30 mg/kg) for 14 weeks, in a mouse model of coronary stenosis, resulted in significant suppression of inflammation and reduced the incidence of coronary artery stenosis of mice in which inflammation and stenosis were induced with *Lactobacillus casei* cell wall extract^8^. Similarly, oral administration of RA (10 mg/kg/day) significantly mitigated the stenosis extent of vein grafts in a rabbit autogenous vein grafts model after 2, 4 and 8 weeks of treatment^9^. Furthermore, Leville et al. reported that oral administration of RA (10 mg/kg/day) decreases cell proliferation, increases apoptosis, and decreases the expression and activity of matrix metalloproteinases in an animal model vein bypass grafting^16,17^. Some years later, Gregory et al. used peripheral bypass graft able to immobilize RA through porous poly membranes in a rat aortic bypass model. Results indicated reduced intimal hyperplasia, percent stenosis, and greater lumen area^10^.

Previous studies have examined the role of RA in reducing neointimal formation after vascular injury. Miano et al. showed that orally administered all-trans-retinoic acid increased the luminal area (by 35% to 37%) after balloon injury in rat carotid arteries^18^. Similarly, DeRose et al. showed inhibition of vessel remodeling as measured by increases in luminal diameter, using 10 mg/kg all-trans retinoic acid^19^. In another study, Wiegman et al. investigated the effect of orally delivered RA (pre-treatment for 3 days before and 28 days after balloon angioplasty) in plaque size and vessel geometry after balloon angioplasty performed on rabbits with focal femoral atherosclerosis. Histomorphometry measurements showed that *per os* administration of RA resulted in larger lumen area. However, no differences in absolute plaque area were observed^20^.

Other studies involved local administration of RA aiming to prevent the adverse effects caused by the higher doses of systematic delivery of RA (mucocutaneous side effects, liver toxicity and abnormalities of serum lipid profiles^12^. Herdeg et al., after performing plaque formation in rabbits right carotid artery and undergoing balloon angioplasty, administered RA locally (10 ml, 10 μM) by using a double-balloon catheter. Results showed a non-significant limitation of restenosis formation 4 weeks after intervention (p = 0.0937)^11^. In another experimental model of restenosis after balloon injury, citrate-based polyester membranes containing retinoic acid, were wrapped around the carotids of rats that had undergone balloon injury. The membranes inhibited neointimal formation after balloon injury and decreased restenosis^21^.

There is evidence in the literature supporting the notion that RA can reduce neointimal formation after balloon injury but it is not known if this effect also applies for stents. In our study for the first time RA was administered locally by drug eluting stents, in order to allow a slow release of RA and therefore induce a longer-lasting action, while avoiding the adverse effects caused by systematic delivery of RA. Stents were implanted in both femoral arteries of rabbits. We tested three different doses of RA, two different polymers, one bare metal stent, everolimus eluting stent (with the same Cobalt-Chrome architecture and the same polymers) and one of the most popular drug eluting stent in the last decade (Xience Pro).

The presence and severity of inflammation was investigated as part of the histological analysis of target arterial segments. Inflammatory responses were expected to be observed in light of the endothelial damage & subsequent healing mechanisms caused by stent implantation that induced mechanical stress on the vascular wall when expanded or apposed, respectively. However, a biocompatible stress material & coating would be expected to inhibit further/chronic inflammation, while the presence of RA might inhibit inflammatory responses further due to its therapeutic effects, if confirmed. For that reason, markers of inflammation discussed in Results and shown in Table 1, were assessed and demonstrated low & expected levels of local inflammation, with minor differences between different device groups. It would be important to emphasise that absence of inflammation is impossible to attain under this established angioplasty protocol—the degree of inflammation detected by immune cell infiltrates, intima growth due to cell migration & proliferation, occasional local micro-angiogenesis and evidence of tissue damage confirms the expected tissue reaction to stent implantation and is considered to be acceptable in light of total lack of evidence suggesting severe/chronic inflammatory responses and host tissue reaction to the implant under investigation, or its RA/polymer coating. This evidence was in support of and concurrent with the overall positive assessment for RA-coated stents included in this study, in terms of safety & biocompatibility.

In the comparative evaluation, differences among the groups in total arterial reaction average score were limited, which translates into limited differences in biocompatibility and performance among the treatments. The histopathological evaluation showed that the total arterial reaction average score was numerically lower in groups with polymer B compared to polymer A in RA drug eluting stents. The difference was mostly associated with less severe vascular wall changes. Histomorphometrically, RA drug eluting stents with polymer B had numerically lower stenosis and intimal mean thickness values when compared to groups with RA drug eluting stents with polymer A. Moreover, stents with polymer A and high dose resulted in larger stenosis and intimal mean thickness values (the difference was statistically significant compared to Xience Pro group regarding stenosis). These results indicate that stents with polymer A were deemed to induce more vascular changes and stenosis than stents with polymer B. Regarding dose–effect, the result from the combination with the lower dose and polymer A was statistically significantly different from the AH group for intimal mean thickness, suggesting a drug delivery type A dose-related increase of the intimal layer thickness. No such dose effect was observed for drug delivery type B and no statistically significant differences were present between BL and BH groups for all measurements.

Groups EF and EG showed less total arterial reaction score than group C (BMS). Histomorphometrically, Group EG was significantly different compared to reference item Group C for Intimal Area, Stenosis and Intimal Mean Thickness. Group EF showed significant difference compared to reference item Group C for Intimal Mean Thickness. Despite the fact that no statistically significant difference between Groups EF and EG was present by histomorphometric analysis, Group EF showed slightly higher total arterial reaction average score than group EG and Group EF showed greater percentage of stenosis when compared to Group EG. This trend aligned with the superiority observed with BL, BM and BH compared to groups AL, AM, AH.

The unstained particles detected within the tunica intima in all groups, except for group C, were associated with scattered macrophages, giant cells and considered to elicit a minimal “foreign-body” reaction. The origin is unknown, however, it is likely debris from the stent polymer or from the implantation procedure. Generally, this was considered a minimal change, but these particles might contribute to the triggering of the local immune response and neointimal proliferation.

Our study has some limitations. First, all stents were implanted in healthy iliac arteries (without atherosclerotic lesions). Second, iliac arteries (elastic-natured) are different from muscular coronary arteries. Third, observation period in our study was 4 weeks. This period was chosen according to current guidelines for a first evaluation of the feasibility and safety^22,23^. Further investigation is needed in order to examine the possible long term effects of RA eluting stents. Finally, we did not measure the RA concentrations in the iliac wall.

## Conclusions

RA, a non-cytotoxic drug, is known to inhibit neointimal formation after balloon injury in various experimental models.

The present research is the first to investigate the safety and efficacy of a RA eluting stent in an experimental model of restenosis. We showed that RA eluting stent implantation is not related with thrombus formation or medial/transmural rupture and has an acceptable degree of stenosis 4 weeks post implantation in a rabbit iliac artery model. The dose of RA and the type of polymer may play a role in biocompatibility of the device. Further investigation is needed in order to clarify the role of RA in vascular injury after DES implantation.

## Methods

The study was carried out in compliance with the ARRIVE guidelines^24^ and the European Union legislation^25^ following the principle of 3Rs (Reduction, Refinement, Replacement) to ensure: a reduction in the number of animals used, the improvement of experimental techniques and their living conditions so that they are kept to a minimum pain or suffering. The experiments were performed at the Biomedical Research Foundation of the Academy of Athens, Greece / Athens. The protocol was approved by the Protocol Evaluation Committee of the Biomedical Research Foundations of the Academy of Athens, Greece and the Directorate of Agricultural and Veterinary Policy of the Prefecture of Attica, Greece, a State authority. All methods were performed in accordance with the relevant National and international guidelines and regulations.

### Stent placement

Endovascular stents were implanted in the iliac arteries of 30 male New Zealand (2.5 to 3.5 kg) rabbits. The rabbit iliac artery model was chosen as a first test for the preclinical evaluation of the RA eluting stents because the size and injury response of rabbit iliac arteries are relatively comparable to human coronary arteries^22,26^. The animals were individually housed in stainless steel cages. Two stents of the same group were implanted in each rabbit, one stent in each iliac artery. Sixty stents were used in total, which were divided in ten groups, six stents per group, three animals per group. The observation period after implantation was 4 weeks.

### Stent implantation procedure

The rabbits were anesthetized by an intramuscular injection of ketamine (35 mg/kg) and xylazine (7 mg/kg) and were monitored by peripheral pulse oximetry during the procedure. We used a published method of percutaneous transauricular endovascular access^27^. In brief, the animals were placed in supine position, and immobilized. Both auricular dorsa were shaved and sterilized with povidone-iodine. A novice trainee and an experienced interventional cardiologist performed the trans-auricular vascular access, elective vessel catheterizations, and stent implantation in the iliac arteries.

For the initial implantations and in order to establish an accurate stent placement, as well as for collecting data required by a parallel study aiming to develop an in silico simulation of the expansion process^28^ optical coherence tomography (OCT) was performed post-procedural in a small number of stents (Ν = 4). The preparation and materials used for the process were slightly modified for those cases that OCT was performed.

The central auricular artery was punctured with a 22-gauge intravenous catheter approximately at the distal half of its subcutaneous course. Then the central needle of the catheter was removed. A 0.018-inch (V18, Boston scientific, MA) hydrophilic guide wire was carefully advanced into the external carotid artery and aortic arch / descending aorta and then into the thoracic aorta. The catheter was removed, and a 3-cm-long incision of the dermis was performed at the point of the initial puncture along the course of the guide wire to the base of the dorsa. In cases where OCT was scheduled to be performed, a 5-F compatible 0.035-inch, slender introducer sheath (Terumo, Tokyo, Japan) was advanced into the external carotid artery after serial step-by-step dilations with the sheath’s own dilator. Repeated over-the-wire dilations were performed in order to remove the tight and narrow peripheral segment of the artery prior to sheath insertion. Then, heparin (100 IU/kg) was administered intra-arterially. Upon access gaining a 5F guiding catheter (Envoy MPC, Codman, USA) was advanced to the distal aorta to get direct negotiation with targeting vessels and an angiography was performed to visually evaluate the vessel diameter and as roadmap for accessing femoral vessels. Following angiography, the wire was exchanged to an 0.014-inch wire (Lotus II, Rontis, Switzerland), and it was advanced to the femoral artery. Finally, the stent was forwarded and placed, a few millimeters after the origin of the iliac artery (10Atm, 20 s). Following the stent implantation, OCT was performed without contrast media but via washing out with saline solution only.

In cases where the OCT was not scheduled, the process was exactly the same as above, up to the point of the introducer sheath use entry. A 4-F, 0.018-inch compatible Radifocus Introducer II sheath (Terumo, Tokyo, Japan) was used to gain the access point and the following activities were the same as described above. Another differentiation was on the guiding catheter where the above referred catheter was replaced with a 4-F angiographic catheter (Impress VER-H, Merit Medical, USA). A final angiography was performed for evaluating the stent apposition, the vascular patency and the presence of possible complications (e.g.: formation of thrombus, presence of intimal flap, a sub-optimally deployed stent etc.). The animals did not receive any antiplatelet medication.

### Type of stents

For the preparation of the drug eluting stents, Cobalt-Chrome bare metal stents (Leader Plus, Rontis, Zug, Switzerland) with dimensions 12 mm length × 2.50 mm diameter were coated with coatings consisting of a mixture of polymer and drug (Table 3). Two different types of polymers were used, polymer “A”, poly (lactic-co-glycolic acid) (PLGA) and polymer “B”, poly lactic acid (PLA). Three different doses of the drug RA were used with polymers “A” and “B” resulting in three groups of stents for each polymer: AL, AM, AH with polymer A and “Low”, “Medium” and “High” dose respectively and BL, BM, BH with polymer B and “Low”, “Medium” and “High” dose respectively. As retinoic acid is light and oxygen sensitive, care was given to protect the drug from degradation during the coating process and storage. The drug was handled under a dim yellow light during preparation of the coating mixtures. After coating and crimping on catheters, the stent systems were packaged in non-transparent aluminum foil packages with moisture and oxygen absorbers. In order to ensure the quantity of the drug on the stents, quality control tests were performed after production. Two groups of stents, namely EF and EG with the same dose of everolimus were prepared with the use of polymers A and B respectively. All coated stents were crimped on Rontis’ Leader Plus stent delivery systems with dimensions 2.5 × 12 (REF: LP20/12).

**Table 3:** The types of stents, compositions of the coatings and the drug doses of the tested items.

Group	Polymer	Drug	Drug dose (μg/12 mm stent)	Dimensions [length (mm) × diameter (mm)]
AL	A (PLGA)	RA	48	2.5 × 12
AM	A (PLGA)	RA	77	2.5 × 12
AH	A (PLGA)	RA	112	2.5 × 12
BL	B (PLA)	RA	48	2.5 × 12
BM	B (PLA)	RA	77	2.5 × 12
BH	B (PLA)	RA	135	2.5 × 12
C	–	–	–	2.5 × 12
D	Fluoropolymer (poly(vinylidene fluoride-*co*-hexafluoropropylene) and poly(*n-*butyl methacrylate)	Everolimus	58	2.5 × 12
EF	A (PLGA)	Everolimus	92	2.5 × 12
EG	B (PLA)	Everolimus	92	2.5 × 12

Due to differences in geometry between the stent types used, the total drug dose in μg is mentioned instead of a dose expressed in drug mass per stent surface in μg/mm^2^.

*A* polymer A, *PLG* poly (lactic-*co*-glycolic acid), *B* polymer B, *PLA* poly lactic acid, *L* low (dose), *M* medium (dose), *H* high (dose), *C* Rontis bare metal stent, *D* Xience Pro everolimus eluting coronary stent system, Abbot vascular, *EF and EG* Rontis everolimus stents with the use of polymers A and B respectively, *RA* retinoic acid.

Rontis Cobalt-Chrome bare metal (uncoated) stents and commercially available Everolimus eluting stents (Xience Pro Everolimus Eluting Coronary Stent System, Abbot Vascular, Santa Clara CA, USA) were also used of the same size (group C and D, respectively). The types of stents, compositions of the coatings and the drug doses of the tested items are included in Table 3.

### Necropsy and histomorphometry

The animals were euthanized with sodium pentobarbitone injection. Necropsies were performed by an experienced veterinary doctor. The treated artery segments were collected and preserved in 10% neutral buffered formalin and transferred for histological processing, image analysis and histopathological evaluation. Histology and morphometry were performed at an independent Good Laboratory Practice certified laboratory (Anapath Services, GmbH, Switzerland) by a board-certified veterinarian pathologist. Samples from implants sites were processed by methyl methacrylate (MMA) resin embedding, sawed by a diamond band saw in transversal sample direction (EXAKT System), ground and polished to a final thickness of approximately 40–60 µm (EXAKT System). One section from each sample was stained with adapted Paragon stain following Standard Operating Procedures. Evaluation of the slides for quality check was performed, and then slides were transferred to the study pathologist for pathology evaluation.

### Evaluation parameters

The parameters evaluated by histopathology examination adapted to the scoring system described by ISO 10993-6:2016 were: arterial reaction [endothelial loss (%), surface (fibrin/platelet thrombus), intima proliferation, smooth muscle in intima proliferation, proteoglycan/collagen], artery inflammation (intima/media, Adventitia), medial smooth muscle cell (SMC) loss [Medial SMC loss (transmural), Medial SMC loss (circumference)], artery cell replacement tissue (proteoglycans, collagen, adventitia), host reaction associated with the implant (polymorphonuclear cells, lymphocytes, plasma cells, macrophages, giant cells, necrosis, fibrosis, peristrut hemorrhage/fibrin accumulation, neovascularization, fatty Infiltrate), elastic lamina (EL) rupture (external EL rupture, internal EL rupture), medial hypertrophy (focal, diffuse). An adapted scoring system, described by ISO 10993-6:2016, was applied. Each parameter scored from 0 to 4 according to the histological findings. Defined by the ISO 10993-6:2016, a score difference between 0.0 to 2.9 is considered no or minimal host reaction, 3.0 to 8.9 slight host reaction, 9.0 to 15.0 moderate host reaction and ≥ 15.1 severe host reaction compared to a reference material.

An Olympus Slideview VS200 slides scanner using an Olympus U-TV1XC camera and 20× objective was used to scan the arterial samples. Quantitative evaluation was performed using Olympus imaging and image analysis software cellSens v1.18.

**Figure 3:**
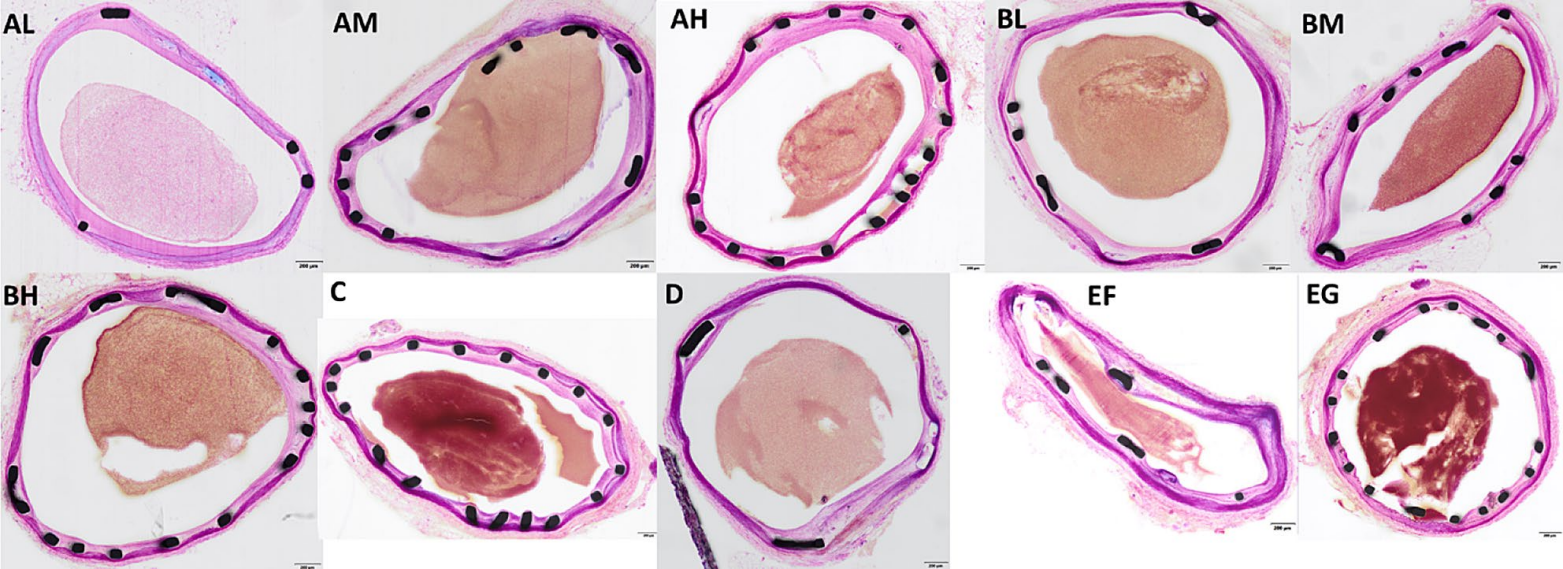
Representative images displaying measurements, one image per treatment group. Paragon, objective ×20. The images show measurement parameters, external elastic lamina (EEL), internal elastic lamina (IEL), lumen, intima and media. Ten approximate equidistant measurements were used for measuring the intimal thickness. *A* Polymer A; *PLG* poly (lactic-*co*-glycolic acid); *B* polymer B; *PLA* poly lactic acid; *L* low (dose); *M* medium (dose), *H* high (dose); *C* Rontis bare metal stent; *D* Xience Pro everolimus eluting coronary stent system, Abbot vascular; *EF and EG* Rontis everolimus stents with the use of polymers A and B respectively. *RA* retinoic acid.

Quantitative evaluation by image analysis on each artery was performed, including the following parameters: Area within external elastic lamina (EEL; µm^2^), area within internal elastic lamina (IEL; µm^2^), lumen (µm^2^), intima (µm^2^) (calculation: IEL-lumen), media (µm^2^) (calculation: EEL − IEL), stenosis (%) [calculation:100 − (100 × Lumen/IEL)], intimal thickness (µm) (average value from 10 approximate equidistant thick measurements). These arithmetic mean values were used for further descriptive statistics. Illustrative images with the measurements, one for each group, are displayed in Fig. 3. As arteries were not flushed during sampling to avoid missing any possible presence of local thrombosis, red blood cells (round anuclear cells) were present in the lumens of several arteries.

### Statistical analysis

Statistical tests were performed using the Prism 8 software (GraphPad, San Diego, CA, USA). Descriptive statistics were used for medial area, intimal area, stenosis and intimal thickness. The Shapiro–Wilk test for normality was performed. When the data followed normal distribution, the comparisons were performed with the unpaired t-test. When data did not follow normal distribution, the Mann–Whitney test was used. A p value less than 0.05 was considered significant.

## Supplementary Information

Supplementary Information.

## Data Availability

The raw data of this manuscript are available as supplemental material.
