# Supplementary material for: Histopathological evaluation of a retinoic acid eluting stent in a rabbit iliac artery model
Source: Sci Rep. 2022 Aug 3;12:13305. doi: 10.1038/s41598-022-16025-5 (PMC9349184; doi:10.1038/s41598-022-16025-5)

**Histopathological evaluation of a retinoic acid eluting stent in a rabbit iliac artery model.**

**Ioanna Samara^1+^, Christos S Katsouras^1,2+^, Arsen Semertzioglou^3^, Athanassios Vratimos^3^, Amalia I Moula^4,5^, Constantinos A Dimitriou^6^, Michail Theofanis^7^, Triantafyllia Papadimitropoulou^4^, Vasileios Bouratzis^2^, Georgia Karanasiou^8^, Dimitrios Fotiadis^8^, Lampros K Michalis^1,2^, Anargyros N Moulas^4^*.**

**^+^**Ioanna Samara and Christos S Katsouras share first authorship

*Corresponding author: Email: [moulas@uth.gr](mailto:moulas@uth.gr)

^1^2^nd^ Department of Cardiology, University Hospital of Ioannina, Ioannina, Greece, ^2^Faculty of Medicine, School of Health Sciences, University of Ioannina, Ioannina, Greece, ^3^Rontis Corporation, Switzerland, ^4^General Department, University of Thessaly, Larissa, Greece, ^5^Faculty of Health Medicine and Life Sciences, University of Maastricht, Maastricht, The Netherlands, ^6^Center for Clinical, Experimental Surgery and Translational Research, Biomedical Research Foundation of the Academy of Athens, Athens, Greece, ^7^Department of interventional radiology, University hospital of Patras, Patras, Greece, ^8^Department of Biomedical Research, Institute of Molecular Biology and Biotechnology, Dept. of Materials Science and Engineering, Unit of Medical Technology and Intelligent Information Systems, University of Ioannina, Ioannina, Greece.

# Supplemental material.

## Supplement I. Individual data from histopathology evaluation

Table 1. Individual sample data. Vascular wall and inflammation findings.

|  |  |  | **Vascular wall findings** | | | | | | **Artery Inflammation** | | |
| --- | --- | --- | --- | --- | --- | --- | --- | --- | --- | --- | --- |
| **Animal** | **Group** | **Iliac Artery Side** | Endothelial loss (%) | Surface (Fibrin/platelet thrombus) | Intima proliferation | Smooth muscle in intima proliferation | Proteoglycan/collagen | **Subtotal (x2)** | Intima/ media | Adventitia | **Subtotal (x2)** |
| 1048 | AL | Right | 1 | 0 | 3 | 2 | 1 | **14** | 1 | 0 | **2** |
| 1048 | AL | Left | 1 | 0 | 3 | 2 | 1 | **14** | 1 | 0 | **2** |
| 1049 | AM | Right | 1 | 0 | 2 | 2 | 0 | **10** | 1 | 0 | **2** |
| 1049 | AM | Left | 1 | 0 | 1 | 1 | 0 | **6** | 1 | 0 | **2** |
| 1050 | AH | Right | 1 | 0 | 2 | 1 | 1 | **10** | 1 | 0 | **2** |
| 1050 | AH | Left | 2 | 0 | 2 | 1 | 1 | **12** | 1 | 0 | **2** |
| 1051 | BL | Right | 1 | 0 | 2 | 2 | 0 | **10** | 2 | 1 | **6** |
| 1051 | BL | Left | 1 | 0 | 2 | 2 | 0 | **10** | 1 | 0 | **2** |
| 1064 | BM | Right | 1 | 0 | 2 | 2 | 0 | **10** | 2 | 1 | **6** |
| 1064 | BM | Left | 0 | 0 | 2 | 2 | 0 | **8** | 1 | 0 | **2** |
| 1067 | C | Right | 1 | 0 | 2 | 2 | 0 | **10** | 1 | 0 | **2** |
| 1067 | C | Left | 1 | 0 | 2 | 2 | 0 | **10** | 2 | 0 | **4** |
| 1068 | AM | Right | 1 | 0 | 2 | 2 | 0 | **10** | 1 | 0 | **2** |
| 1068 | AM | Left | 0 | 0 | 3 | 3 | 0 | **12** | 1 | 0 | **2** |
| 1069 | BH | Right | 0 | 0 | 2 | 2 | 0 | **8** | 2 | 0 | **4** |
| 1069 | BH | Left | 0 | 0 | 3 | 3 | 0 | **12** | 1 | 0 | **2** |
| 1070 | EF | Right | 0 | 0 | 2 | 2 | 0 | **8** | 1 | 0 | **2** |
| 1070 | EF | Left | 0 | 0 | 1 | 1 | 0 | **4** | 2 | 0 | **4** |
| 1071 | EG | Right | 1 | 0 | 2 | 1 | 1 | **10** | 1 | 0 | **2** |
| 1071 | EG | Left | 1 | 0 | 2 | 2 | 0 | **10** | 1 | 0 | **2** |
| 1072 | AL | Right | 1 | 0 | 2 | 2 | 0 | **10** | 1 | 0 | **2** |
| 1072 | AL | Left | 0 | 0 | 2 | 2 | 0 | **8** | 1 | 0 | **2** |
| 1074 | AH | Aorta | 1 | 0 | 2 | 2 | 0 | **10** | 1 | 0 | **2** |
| 1074 | AH | Left | 1 | 0 | 3 | 3 | 0 | **14** | 1 | 0 | **2** |
| 1075 | BL | Right | 0 | 0 | 2 | 1 | 1 | **8** | 1 | 0 | **2** |
| 1075 | BL | Left | 1 | 0 | 2 | 1 | 1 | **10** | 0 | 0 | **0** |
| 1079 | D | Right | 1 | 0 | 1 | 1 | 0 | **6** | 1 | 0 | **2** |
| 1079 | D | Left | 1 | 0 | 2 | 1 | 1 | **10** | 1 | 0 | **2** |
| 1080 | BH | Aorta | 1 | 0 | 2 | 2 | 0 | **10** | 1 | 0 | **2** |
| 1080 | BH | Left | 0 | 0 | 2 | 1 | 1 | **8** | 1 | 0 | **2** |
| 1081 | C | Right | 1 | 0 | 2 | 2 | 0 | **10** | 1 | 0 | **2** |
| 1081 | C | Left | 0 | 0 | 3 | 1 | 1 | **10** | 1 | 0 | **2** |
| 1082 | D | Right | 1 | 0 | 1 | 0 | 1 | **6** | 2 | 0 | **4** |
| 1082 | D | Left | 1 | 0 | 2 | 2 | 0 | **10** | 1 | 0 | **2** |
| 1083 | EF | Right | 2 | 0 | 2 | 2 | 0 | **12** | 2 | 2 | **8** |
| 1083 | EF | Left | 2 | 0 | 2 | 2 | 0 | **12** | 1 | 0 | **2** |
| 1084 | EG | Right | 2 | 0 | 1 | 1 | 0 | **8** | 1 | 0 | **2** |
| 1084 | EG | Left | 2 | 0 | 1 | 1 | 0 | **8** | 1 | 0 | **2** |
| 1085 | AL | Right | 1 | 0 | 2 | 2 | 0 | **10** | 1 | 0 | **2** |
| 1085 | AL | Left | 0 | 0 | 2 | 2 | 0 | **8** | 1 | 0 | **2** |
| 1086 | AM | Right | 0 | 0 | 3 | 2 | 1 | **12** | 1 | 0 | **2** |
| 1086 | AM | Left | 0 | 0 | 2 | 2 | 0 | **8** | 1 | 0 | **2** |
| 1087 | AH | Right | 1 | 0 | 3 | 2 | 1 | **14** | 2 | 0 | **4** |
| 1087 | AH | Aorta | 2 | 0 | 3 | 2 | 1 | **16** | 2 | 0 | **4** |
| 1088 | BL | Right | 0 | 0 | 2 | 1 | 1 | **8** | 1 | 0 | **2** |
| 1088 | BL | Left | 1 | 0 | 3 | 2 | 1 | **14** | 1 | 0 | **2** |
| 1089 | BM | Right | 0 | 0 | 2 | 2 | 0 | **8** | 1 | 0 | **2** |
| 1089 | BM | Left | 0 | 0 | 1 | 1 | 0 | **4** | 1 | 0 | **2** |
| 1090 | BH | Right | 0 | 0 | 3 | 2 | 1 | **12** | 1 | 0 | **2** |
| 1090 | BH | Left | 0 | 0 | 2 | 2 | 0 | **8** | 1 | 0 | **2** |
| 1091 | C | Right | 0 | 0 | 2 | 1 | 1 | **8** | 1 | 0 | **2** |
| 1091 | C | Left | 1 | 0 | 3 | 2 | 1 | **14** | 1 | 0 | **2** |
| 1092 | D | Right | 0 | 0 | 1 | 1 | 0 | **4** | 1 | 0 | **2** |
| 1092 | D | Left | 1 | 0 | 2 | 1 | 1 | **10** | 1 | 0 | **2** |
| 1093 | EF | Right | 1 | 0 | 1 | 1 | 0 | **6** | 1 | 0 | **2** |
| 1093 | EF | Left | 1 | 0 | 1 | 1 | 0 | **6** | 1 | 0 | **2** |
| 1094 | EG | Right | 1 | 0 | 1 | 1 | 0 | **6** | 1 | 0 | **2** |
| 1094 | EG | Left | 1 | 0 | 2 | 2 | 0 | **10** | 1 | 0 | **2** |

Table 2. Individual sample data. Findings within the tunica media smooth muscle.

|  |  |  | **Artery Medial smooth muscle cell (SMC) loss** | | | **Artery Medial smooth muscle cell replacement tissue** | | | | **Artery Medial hypertrophy** | | |
| --- | --- | --- | --- | --- | --- | --- | --- | --- | --- | --- | --- | --- |
| **Animal** | **Group** | **Iliac Artery Side** | Medial SMC loss (transmural) | Medial SMC loss (circumference) | **Subtotal (x2)** | Proteoglycans | Collagen | Adventitia | **Subtotal** | Media hypertrophy, focal | Media hypertrophy, diffuse | **Subtotal (x2)** |
| 1048 | AL | Right | 0 | 0 | **0** | 0 | 0 | 0 | **0** | 0 | 0 | **0** |
| 1048 | AL | Left | 0 | 0 | **0** | 0 | 0 | 0 | **0** | 0 | 0 | **0** |
| 1049 | AM | Right | 0 | 0 | **0** | 0 | 0 | 0 | **0** | 0 | 0 | **0** |
| 1049 | AM | Left | 0 | 0 | **0** | 0 | 0 | 0 | **0** | 0 | 0 | **0** |
| 1050 | AH | Right | 0 | 0 | **0** | 0 | 0 | 0 | **0** | 0 | 0 | **0** |
| 1050 | AH | Left | 0 | 0 | **0** | 0 | 0 | 0 | **0** | 0 | 0 | **0** |
| 1051 | BL | Right | 0 | 0 | **0** | 0 | 0 | 0 | **0** | 0 | 0 | **0** |
| 1051 | BL | Left | 0 | 0 | **0** | 0 | 0 | 0 | **0** | 0 | 0 | **0** |
| 1064 | BM | Right | 0 | 0 | **0** | 0 | 0 | 0 | **0** | 0 | 0 | **0** |
| 1064 | BM | Left | 0 | 0 | **0** | 0 | 0 | 0 | **0** | 0 | 0 | **0** |
| 1067 | C | Right | 0 | 0 | **0** | 0 | 0 | 0 | **0** | 0 | 0 | **0** |
| 1067 | C | Left | 0 | 0 | **0** | 0 | 0 | 0 | **0** | 0 | 0 | **0** |
| 1068 | AM | Right | 0 | 0 | **0** | 0 | 0 | 0 | **0** | 0 | 0 | **0** |
| 1068 | AM | Left | 0 | 0 | **0** | 0 | 0 | 0 | **0** | 0 | 0 | **0** |
| 1069 | BH | Right | 0 | 0 | **0** | 0 | 0 | 0 | **0** | 0 | 0 | **0** |
| 1069 | BH | Left | 1 | 0 | **2** | 0 | 0 | 0 | **0** | 0 | 0 | **0** |
| 1070 | EF | Right | 1 | 0 | **2** | 0 | 0 | 0 | **0** | 0 | 0 | **0** |
| 1070 | EF | Left | 0 | 0 | **0** | 0 | 0 | 0 | **0** | 0 | 0 | **0** |
| 1071 | EG | Right | 0 | 0 | **0** | 0 | 0 | 0 | **0** | 0 | 0 | **0** |
| 1071 | EG | Left | 0 | 0 | **0** | 0 | 0 | 0 | **0** | 0 | 0 | **0** |
| 1072 | AL | Right | 0 | 0 | **0** | 0 | 0 | 0 | **0** | 0 | 0 | **0** |
| 1072 | AL | Left | 0 | 0 | **0** | 0 | 0 | 0 | **0** | 0 | 0 | **0** |
| 1074 | AH | Aorta | 0 | 0 | **0** | 0 | 0 | 0 | **0** | 0 | 0 | **0** |
| 1074 | AH | Left | 0 | 0 | **0** | 0 | 0 | 0 | **0** | 0 | 0 | **0** |
| 1075 | BL | Right | 0 | 0 | **0** | 0 | 0 | 0 | **0** | 0 | 0 | **0** |
| 1075 | BL | Left | 0 | 0 | **0** | 0 | 0 | 0 | **0** | 0 | 0 | **0** |
| 1079 | D | Right | 0 | 0 | **0** | 0 | 0 | 0 | **0** | 0 | 0 | **0** |
| 1079 | D | Left | 0 | 0 | **0** | 0 | 0 | 0 | **0** | 0 | 0 | **0** |
| 1080 | BH | Aorta | 0 | 0 | **0** | 0 | 0 | 0 | **0** | 0 | 0 | **0** |
| 1080 | BH | Left | 0 | 0 | **0** | 0 | 0 | 0 | **0** | 0 | 0 | **0** |
| 1081 | C | Right | 0 | 0 | **0** | 0 | 0 | 0 | **0** | 0 | 0 | **0** |
| 1081 | C | Left | 0 | 0 | **0** | 0 | 0 | 0 | **0** | 0 | 0 | **0** |
| 1082 | D | Right | 1 | 0 | **2** | 0 | 0 | 0 | **0** | 0 | 0 | **0** |
| 1082 | D | Left | 0 | 0 | **0** | 0 | 0 | 0 | **0** | 0 | 0 | **0** |
| 1083 | EF | Right | 0 | 0 | **0** | 0 | 0 | 0 | **0** | 0 | 0 | **0** |
| 1083 | EF | Left | 0 | 0 | **0** | 0 | 0 | 0 | **0** | 0 | 0 | **0** |
| 1084 | EG | Right | 0 | 0 | **0** | 0 | 0 | 0 | **0** | 0 | 0 | **0** |
| 1084 | EG | Left | 0 | 0 | **0** | 0 | 0 | 0 | **0** | 0 | 0 | **0** |
| 1085 | AL | Right | 0 | 0 | **0** | 0 | 0 | 0 | **0** | 0 | 0 | **0** |
| 1085 | AL | Left | 0 | 0 | **0** | 0 | 0 | 0 | **0** | 0 | 0 | **0** |
| 1086 | AM | Right | 0 | 0 | **0** | 0 | 0 | 0 | **0** | 0 | 0 | **0** |
| 1086 | AM | Left | 0 | 0 | **0** | 0 | 0 | 0 | **0** | 0 | 0 | **0** |
| 1087 | AH | Right | 0 | 0 | **0** | 0 | 0 | 0 | **0** | 0 | 0 | **0** |
| 1087 | AH | Aorta | 0 | 0 | **0** | 0 | 0 | 0 | **0** | 0 | 0 | **0** |
| 1088 | BL | Right | 0 | 0 | **0** | 0 | 0 | 0 | **0** | 0 | 0 | **0** |
| 1088 | BL | Left | 0 | 0 | **0** | 0 | 0 | 0 | **0** | 0 | 0 | **0** |
| 1089 | BM | Right | 0 | 0 | **0** | 0 | 0 | 0 | **0** | 0 | 0 | **0** |
| 1089 | BM | Left | 0 | 0 | **0** | 0 | 0 | 0 | **0** | 0 | 0 | **0** |
| 1090 | BH | Right | 0 | 0 | **0** | 0 | 0 | 0 | **0** | 0 | 0 | **0** |
| 1090 | BH | Left | 0 | 0 | **0** | 0 | 0 | 0 | **0** | 0 | 0 | **0** |
| 1091 | C | Right | 0 | 0 | **0** | 0 | 0 | 0 | **0** | 0 | 0 | **0** |
| 1091 | C | Left | 0 | 0 | **0** | 0 | 0 | 0 | **0** | 0 | 0 | **0** |
| 1092 | D | Right | 0 | 0 | **0** | 0 | 0 | 0 | **0** | 0 | 0 | **0** |
| 1092 | D | Left | 0 | 0 | **0** | 0 | 0 | 0 | **0** | 0 | 0 | **0** |
| 1093 | EF | Right | 0 | 0 | **0** | 0 | 0 | 0 | **0** | 0 | 0 | **0** |
| 1093 | EF | Left | 0 | 0 | **0** | 0 | 0 | 0 | **0** | 0 | 0 | **0** |
| 1094 | EG | Right | 0 | 0 | **0** | 0 | 0 | 0 | **0** | 0 | 0 | **0** |
| 1094 | EG | Left | 0 | 0 | **0** | 0 | 0 | 0 | **0** | 0 | 0 | **0** |

Table 3. Individual sample data. Elastic lamina rupture, host reaction associated with the implant and Total arterial reaction

|  |  |  | **Artery Lamina elastic rupture** | | | **Host reaction associated with the implant** | | | | | | | | | | | | | |  |
| --- | --- | --- | --- | --- | --- | --- | --- | --- | --- | --- | --- | --- | --- | --- | --- | --- | --- | --- | --- | --- |
| **Animal** | **Group** | **Iliac Artery Side** | EEL rupture | IEL rupture | **Subtotal (x2)** | Polymorphonuclear cells | Lymphocytes | Plasma cells | Macrophages | Giant cells | Necrosis | **Subtotal (x2)** | Fibrosis | Fatty Infiltrate | Peristrut haemorrhage | Neovascularization | **Subtotal** | **Subtotal Host Reaction** | **TOTAL ARTERIAL REACTION** | Stainless particles |
| 1048 | AL | Right | 0 | 1 | **2** | 0 | 1 | 0 | 1 | 1 | 0 | **6** | 0 | 0 | 1 | 0 | **2** | **8** | **26** | 1 |
| 1048 | AL | Left | 0 | 2 | **4** | 1 | 1 | 0 | 0 | 1 | 0 | **6** | 0 | 0 | 3 | 0 | **3** | **9** | **29** | 1 |
| 1049 | AM | Right | 0 | 1 | **2** | 1 | 1 | 0 | 1 | 1 | 0 | **8** | 0 | 0 | 1 | 0 | **1** | **9** | **23** | 1 |
| 1049 | AM | Left | 0 | 2 | **4** | 0 | 1 | 0 | 0 | 1 | 0 | **4** | 0 | 0 | 2 | 0 | **2** | **6** | **18** | 1 |
| 1050 | AH | Right | 0 | 1 | **2** | 1 | 0 | 0 | 0 | 0 | 0 | **2** | 0 | 0 | 1 | 0 | **1** | **3** | **17** | 0 |
| 1050 | AH | Left | 0 | 1 | **2** | 1 | 1 | 0 | 1 | 0 | 0 | **6** | 0 | 0 | 1 | 1 | **2** | **8** | **24** | 0 |
| 1051 | BL | Right | 0 | 2 | **4** | 0 | 0 | 0 | 2 | 1 | 0 | **6** | 0 | 0 | 1 | 0 | **1** | **7** | **27** | 0 |
| 1051 | BL | Left | 1 | 4 | **10** | 1 | 0 | 0 | 1 | 1 | 0 | **6** | 0 | 0 | 1 | 1 | **2** | **8** | **30** | 2 |
| 1064 | BM | Right | 0 | 2 | **4** | 1 | 1 | 0 | 1 | 1 | 0 | **8** | 0 | 0 | 0 | 0 | **0** | **8** | **28** | 0 |
| 1064 | BM | Left | 0 | 2 | **4** | 0 | 0 | 0 | 1 | 1 | 0 | **4** | 0 | 0 | 0 | 0 | **0** | **4** | **18** | 0 |
| 1067 | C | Right | 0 | 1 | **2** | 1 | 0 | 0 | 0 | 1 | 0 | **4** | 2 | 0 | 1 | 0 | **3** | **7** | **21** | 0 |
| 1067 | C | Left | 0 | 1 | **2** | 1 | 1 | 0 | 2 | 1 | 0 | **10** | 0 | 0 | 1 | 0 | **1** | **11** | **27** | 0 |
| 1068 | AM | Right | 0 | 1 | **2** | 1 | 1 | 0 | 1 | 1 | 0 | **8** | 0 | 0 | 4 | 1 | **5** | **13** | **27** | 1 |
| 1068 | AM | Left | 0 | 0 | **0** | 1 | 1 | 1 | 2 | 1 | 0 | **12** | 0 | 0 | 0 | 0 | **0** | **12** | **26** | 2 |
| 1069 | BH | Right | 0 | 0 | **0** | 1 | 0 | 1 | 1 | 1 | 0 | **8** | 0 | 0 | 0 | 0 | **0** | **8** | **20** | 1 |
| 1069 | BH | Left | 2 | 2 | **8** | 1 | 1 | 0 | 1 | 1 | 0 | **8** | 0 | 0 | 0 | 0 | **0** | **8** | **32** | 2 |
| 1070 | EF | Right | 0 | 1 | **2** | 0 | 0 | 0 | 1 | 1 | 0 | **4** | 0 | 0 | 2 | 0 | **2** | **6** | **20** | 0 |
| 1070 | EF | Left | 0 | 2 | **4** | 1 | 1 | 0 | 1 | 1 | 0 | **8** | 0 | 0 | 2 | 0 | **2** | **10** | **22** | 0 |
| 1071 | EG | Right | 0 | 0 | **0** | 1 | 1 | 0 | 1 | 1 | 0 | **8** | 0 | 0 | 0 | 1 | **1** | **9** | **21** | 1 |
| 1071 | EG | Left | 0 | 1 | **2** | 1 | 1 | 0 | 1 | 1 | 0 | **8** | 0 | 0 | 0 | 0 | **0** | **8** | **22** | 2 |
| 1072 | AL | Right | 0 | 0 | **0** | 0 | 0 | 0 | 1 | 1 | 0 | **4** | 0 | 0 | 1 | 0 | **1** | **5** | **17** | 0 |
| 1072 | AL | Left | 0 | 0 | **0** | 1 | 0 | 0 | 1 | 1 | 0 | **6** | 0 | 0 | 0 | 0 | **0** | **6** | **16** | 1 |
| 1074 | AH | Aorta | 0 | 1 | **2** | 1 | 1 | 0 | 0 | 0 | 0 | **4** | 1 | 0 | 0 | 0 | **1** | **5** | **19** | 1 |
| 1074 | AH | Left | 0 | 2 | **4** | 0 | 0 | 0 | 1 | 0 | 0 | **2** | 0 | 0 | 1 | 0 | **1** | **3** | **23** | 1 |
| 1075 | BL | Right | 0 | 0 | **0** | 0 | 0 | 0 | 0 | 1 | 0 | **2** | 0 | 0 | 0 | 1 | **1** | **3** | **13** | 2 |
| 1075 | BL | Left | 0 | 0 | **0** | 1 | 1 | 0 | 1 | 1 | 0 | **8** | 1 | 0 | 1 | 1 | **3** | **11** | **21** | 0 |
| 1079 | D | Right | 0 | 0 | **0** | 0 | 1 | 0 | 0 | 1 | 0 | **4** | 0 | 0 | 0 | 0 | **0** | **4** | **12** | 0 |
| 1079 | D | Left | 0 | 0 | **0** | 1 | 1 | 0 | 1 | 1 | 0 | **8** | 0 | 0 | 2 | 0 | **2** | **10** | **22** | 1 |
| 1080 | BH | Aorta | 0 | 1 | **2** | 1 | 0 | 0 | 1 | 1 | 0 | **6** | 1 | 0 | 0 | 0 | **1** | **7** | **21** | 2 |
| 1080 | BH | Left | 0 | 1 | **2** | 0 | 1 | 1 | 1 | 1 | 0 | **8** | 0 | 0 | 1 | 0 | **1** | **9** | **21** | 2 |
| 1081 | C | Right | 0 | 2 | **4** | 1 | 1 | 0 | 1 | 1 | 0 | **8** | 1 | 0 | 0 | 0 | **1** | **9** | **25** | 0 |
| 1081 | C | Left | 0 | 1 | **2** | 1 | 1 | 0 | 1 | 1 | 0 | **8** | 1 | 0 | 0 | 1 | **2** | **10** | **24** | 0 |
| 1082 | D | Right | 1 | 2 | **6** | 1 | 1 | 0 | 1 | 1 | 0 | **8** | 2 | 0 | 1 | 0 | **3** | **11** | **29** | 0 |
| 1082 | D | Left | 1 | 1 | **4** | 1 | 1 | 1 | 1 | 1 | 0 | **10** | 0 | 0 | 1 | 0 | **1** | **11** | **27** | 1 |
| 1083 | EF | Right | 1 | 1 | **4** | 2 | 1 | 0 | 1 | 0 | 0 | **8** | 1 | 0 | 2 | 0 | **3** | **11** | **35** | 1 |
| 1083 | EF | Left | 0 | 1 | **2** | 1 | 1 | 0 | 1 | 1 | 0 | **8** | 0 | 0 | 1 | 0 | **1** | **9** | **25** | 0 |
| 1084 | EG | Right | 0 | 2 | **4** | 1 | 2 | 0 | 1 | 1 | 0 | **10** | 0 | 0 | 2 | 0 | **2** | **12** | **26** | 0 |
| 1084 | EG | Left | 1 | 0 | **2** | 1 | 0 | 0 | 1 | 0 | 0 | **4** | 0 | 0 | 1 | 0 | **1** | **5** | **17** | 0 |
| 1085 | AL | Right | 1 | 2 | **6** | 1 | 1 | 0 | 1 | 1 | 0 | **8** | 0 | 0 | 2 | 0 | **2** | **10** | **28** | 0 |
| 1085 | AL | Left | 0 | 1 | **2** | 1 | 1 | 0 | 1 | 1 | 0 | **8** | 0 | 0 | 0 | 0 | **0** | **8** | **20** | 1 |
| 1086 | AM | Right | 0 | 1 | **2** | 1 | 0 | 0 | 0 | 1 | 0 | **4** | 1 | 0 | 2 | 0 | **3** | **7** | **23** | 1 |
| 1086 | AM | Left | 0 | 2 | **4** | 0 | 0 | 0 | 1 | 1 | 0 | **4** | 0 | 0 | 0 | 0 | **0** | **4** | **18** | 1 |
| 1087 | AH | Right | 0 | 0 | **0** | 1 | 1 | 0 | 1 | 1 | 0 | **8** | 1 | 0 | 3 | 0 | **4** | **12** | **30** | 2 |
| 1087 | AH | Aorta | 0 | 0 | **0** | 0 | 1 | 0 | 1 | 1 | 0 | **6** | 2 | 0 | 0 | 2 | **4** | **10** | **30** | 2 |
| 1088 | BL | Right | 0 | 0 | **0** | 1 | 0 | 0 | 1 | 1 | 0 | **6** | 0 | 0 | 0 | 0 | **0** | **6** | **16** | 3 |
| 1088 | BL | Left | 0 | 0 | **0** | 1 | 0 | 0 | 0 | 1 | 0 | **4** | 0 | 0 | 1 | 0 | **1** | **5** | **21** | 2 |
| 1089 | BM | Right | 0 | 0 | **0** | 1 | 0 | 0 | 1 | 1 | 0 | **6** | 0 | 0 | 0 | 0 | **0** | **6** | **16** | 3 |
| 1089 | BM | Left | 0 | 0 | **0** | 1 | 1 | 0 | 1 | 1 | 0 | **8** | 0 | 0 | 0 | 0 | **0** | **8** | **14** | 2 |
| 1090 | BH | Right | 0 | 1 | **2** | 1 | 1 | 0 | 1 | 1 | 0 | **8** | 0 | 0 | 0 | 0 | **0** | **8** | **24** | 2 |
| 1090 | BH | Left | 0 | 0 | **0** | 1 | 0 | 0 | 1 | 1 | 0 | **6** | 0 | 0 | 0 | 0 | **0** | **6** | **16** | 1 |
| 1091 | C | Right | 0 | 1 | **2** | 1 | 1 | 0 | 1 | 1 | 0 | **8** | 0 | 0 | 1 | 0 | **1** | **9** | **21** | 0 |
| 1091 | C | Left | 0 | 1 | **2** | 1 | 1 | 0 | 1 | 1 | 0 | **8** | 0 | 0 | 1 | 0 | **1** | **9** | **27** | 0 |
| 1092 | D | Right | 0 | 0 | **0** | 1 | 1 | 0 | 1 | 1 | 0 | **8** | 1 | 0 | 0 | 0 | **1** | **9** | **15** | 0 |
| 1092 | D | Left | 0 | 1 | **2** | 1 | 1 | 0 | 1 | 0 | 0 | **6** | 1 | 0 | 1 | 0 | **2** | **8** | **22** | 1 |
| 1093 | EF | Right | 0 | 0 | **0** |  | 1 | 0 | 1 | 1 | 0 | **6** | 0 | 0 | 1 | 0 | **1** | **7** | **15** | 0 |
| 1093 | EF | Left | 0 | 1 | **2** | 1 | 1 | 0 | 0 | 1 | 0 | **6** | 0 | 0 | 1 | 0 | **1** | **7** | **17** | 0 |
| 1094 | EG | Right | 0 | 1 | **2** | 1 | 0 | 0 | 0 | 1 | 0 | **4** | 0 | 0 | 1 | 0 | **1** | **5** | **15** | 1 |
| 1094 | EG | Left | 0 | 1 | **2** | 0 | 1 | 0 | 1 | 1 | 0 | **6** | 0 | 0 | 2 | 0 | **2** | **8** | **22** | 2 |

## Supplement II. Individual data from histomorphometry

Table 4: **Group AL**, Single Values per Stent Type and Vessel. Area within EEL, Area within IEL, Lumen, Media and Intima (µm^2^). Stenosis (%), Intimal Mean Thickness (µm).

| **Animal no.** | **Side** | **Artery** | **Stent type** | **Area Within EEL (µm^2^)  Measured** | **Area Within IEL (µm^2^)  Measured** | **Lumen Area (µm^2^)  Measured** | **Media Area (µm^2^) Calculated** | **Intima Area (µm^2^) Calculated** | **% Stenosis Calculated** | **Intimal Mean  Thickness (µm)** |
| --- | --- | --- | --- | --- | --- | --- | --- | --- | --- | --- |
| 1048 | Left | Iliac | AL | 2176728,51 | 1777942,10 | 1108235,97 | 398786,41 | 669706,13 | 37,67 | 103,25 |
| 1048 | Right |  | AL | 2602275,16 | 2306422,09 | 1484445,88 | 295853,07 | 821976,20 | 35,64 | 127,04 |
| 1072 | Left |  | AL | 4008452,95 | 3677089,52 | 3258804,80 | 331363,42 | 418284,73 | 11,38 | 50,95 |
| 1072 | Right |  | AL | 2429993,92 | 2066021,07 | 1844554,83 | 363972,85 | 221466,24 | 10,72 | 46,60 |
| 1085 | Left |  | AL | 2847967,54 | 2534453,26 | 2169911,39 | 313514,28 | 364541,87 | 14,38 | 54,75 |
| 1085 | Right |  | AL | 2353819,04 | 2127798,60 | 1798905,80 | 226020,44 | 328892,80 | 15,46 | 46,15 |

Table 5: **Group AM**, Single Values per Stent Type and Vessel. Area within EEL, Area within IEL, Lumen, Media and Intima (µm^2^). Stenosis (%), Intimal Mean Thickness (µm).

| **Animal no.** | **Side** | **Artery** | **Stent type** | **Area Within EEL (µm^2^)  Measured** | **Area Within IEL (µm^2^)  Measured** | **Lumen Area (µm^2^)  Measured** | **Media Area (µm^2^) Calculated** | **Intima Area (µm^2^) Calculated** | **% Stenosis Calculated** | **Intimal Mean  Thickness (µm)** |
| --- | --- | --- | --- | --- | --- | --- | --- | --- | --- | --- |
| 1049 | Left | Iliac | AM | 3189027,08 | 2916559,91 | 2545993,24 | 272467,17 | 370566,67 | 12,71 | 36,77 |
| 1049 | Right |  | AM | 2969813,66 | 2683035,46 | 2345251,81 | 286778,20 | 337783,65 | 12,59 | 67,18 |
| 1068 | Left |  | AM | 3747800,34 | 3518079,85 | 2577125,61 | 229720,49 | 940954,24 | 26,75 | 143,19 |
| 1068 | Right |  | AM | 3227459,02 | 2911649,68 | 2278259,68 | 315809,34 | 633390,00 | 21,75 | 75,70 |
| 1086 | Left |  | AM | 3121820,14 | 2726004,82 | 2267010,10 | 395815,32 | 458994,72 | 16,84 | 80,08 |
| 1086 | Right |  | AM | 1762181,17 | 1333257,91 | 779798,62 | 428923,25 | 553459,29 | 41,51 | 96,65 |

Table 6: **Group AH**, Single Values per Stent Type and Vessel. Area within EEL, Area within IEL, Lumen, Media and Intima (µm^2^). Stenosis (%), Intimal Mean Thickness (µm).

| **Animal no.** | **Side** | **Artery** | **Stent type** | **Area Within EEL (µm^2^)  Measured** | **Area Within IEL (µm^2^)  Measured** | **Lumen Area (µm^2^)  Measured** | **Media Area (µm^2^) Calculated** | **Intima Area (µm^2^) Calculated** | **% Stenosis Calculated** | **Intimal Mean  Thickness (µm)** |
| --- | --- | --- | --- | --- | --- | --- | --- | --- | --- | --- |
| 1050 | Left | Iliac | AH | 3749585,70 | 3410885,56 | 2797138,20 | 338700,14 | 613747,36 | 17,99 | 109,24 |
| 1050 | Right | Iliac | AH | 3428224,07 | 3024576,15 | 2553512,97 | 403647,92 | 471063,18 | 15,57 | 100,09 |
| 1074 | Left | Iliac | AH | 4172653,68 | 3383119,04 | 2651996,74 | 789534,64 | 731122,30 | 21,61 | 110,56 |
| 1074 | Right | Aorta | AH | 1854295,39 | 1318166,38 | 867416,24 | 536129,01 | 450750,14 | 34,20 | 92,66 |
| 1087 | Left | Aorta | AH | 2424293,40 | 1940190,77 | 1180267,93 | 484102,62 | 759922,85 | 39,17 | 105,63 |
| 1087 | Right | Iliac | AH | 2989186,44 | 2767782,81 | 1970543,39 | 221403,64 | 797239,42 | 28,80 | 112,45 |

Table 7: **Group BL**, Single Values per Stent Type and Vessel. Area within EEL, Area within IEL, Lumen, Media and Intima (µm^2^). Stenosis (%), Intimal Mean Thickness (µm).

| **Animal no.** | **Side** | **Artery** | **Stent type** | **Area Within EEL (µm^2^)  Measured** | **Area Within IEL (µm^2^)  Measured** | **Lumen Area (µm^2^)  Measured** | **Media Area (µm^2^) Calculated** | **Intima Area (µm^2^) Calculated** | **% Stenosis Calculated** | **Intimal Mean  Thickness (µm)** |
| --- | --- | --- | --- | --- | --- | --- | --- | --- | --- | --- |
| 1051 | Left | Iliac | BL | 3253119,09 | 3052444,26 | 2509919,53 | 200674,83 | 542524,73 | 17,77 | 82,53 |
| 1051 | Right |  | BL | 2250490,84 | 1880712,71 | 1576405,34 | 369778,12 | 304307,37 | 16,18 | 54,89 |
| 1075 | Left |  | BL | 3358156,44 | 2845790,41 | 2471601,39 | 512366,03 | 374189,02 | 13,15 | 66,24 |
| 1075 | Right |  | BL | 3548189,64 | 3158856,86 | 2737026,52 | 389332,78 | 421830,34 | 13,35 | 72,36 |
| 1088 | Left |  | BL | 3061863,10 | 2788557,76 | 2204194,47 | 273305,34 | 584363,29 | 20,96 | 107,23 |
| 1088 | Right |  | BL | 3208529,42 | 2931343,06 | 2517123,48 | 277186,37 | 414219,58 | 14,13 | 70,91 |

Table 8: **Group BM**, Single Values per Stent Type and Vessel. Area within EEL, Area within IEL, Lumen, Media and Intima (µm^2^). Stenosis (%), Intimal Mean Thickness (µm).

| **Animal no.** | **Side** | **Artery** | **Stent type** | **Area Within EEL (µm^2^)  Measured** | **Area Within IEL (µm^2^)  Measured** | **Lumen Area (µm^2^)  Measured** | **Media Area (µm^2^) Calculated** | **Intima Area (µm^2^) Calculated** | **% Stenosis Calculated** | **Intimal Mean  Thickness (µm)** |
| --- | --- | --- | --- | --- | --- | --- | --- | --- | --- | --- |
| 1064 | Left | Iliac | BM | 1960455,10 | 1726657,98 | 1041789,67 | 233797,13 | 684868,31 | 39,66 | 136,84 |
| 1064 | Right |  | BM | 3110466,85 | 2743693,31 | 2078038,73 | 366773,54 | 665654,58 | 24,26 | 111,86 |
| 1089 | Left |  | BM | 4120529,98 | 3665302,28 | 3331445,42 | 455227,70 | 333856,86 | 9,11 | 47,38 |
| 1089 | Right |  | BM | 3551089,67 | 3208863,39 | 2759602,67 | 342226,28 | 449260,72 | 14,00 | 65,42 |

Table 9: **Group BH**, Single Values per Stent Type and Vessel. Area within EEL, Area within IEL, Lumen, Media and Intima (µm^2^). Stenosis (%), Intimal Mean Thickness (µm).

| **Animal no.** | **Side** | **Artery** | **Stent type** | **Area Within EEL (µm^2^)  Measured** | **Area Within IEL (µm^2^)  Measured** | **Lumen Area (µm^2^)  Measured** | **Media Area (µm^2^) Calculated** | **Intima Area (µm^2^) Calculated** | **% Stenosis Calculated** | **Intimal Mean  Thickness (µm)** |
| --- | --- | --- | --- | --- | --- | --- | --- | --- | --- | --- |
| 1069 | Left | Iliac | BH | 3542907,39 | 3235161,39 | 2659902,05 | 307746,01 | 575259,34 | 17,78 | 92,30 |
| 1069 | Right | Iliac | BH | 3866009,35 | 3593088,17 | 2951574,30 | 272921,18 | 641513,87 | 17,85 | 65,85 |
| 1080 | Left | Iliac | BH | 2962065,22 | 2319285,20 | 2061574,45 | 642780,02 | 257710,75 | 11,11 | 37,24 |
| 1080 | Right | Aorta | BH | 3036908,65 | 2829863,17 | 2249316,65 | 207045,47 | 580546,52 | 20,52 | 96,43 |
| 1090 | Left | Iliac | BH | 2915604,76 | 2464595,51 | 2080930,13 | 451009,25 | 383665,38 | 15,57 | 56,38 |
| 1090 | Right | Iliac | BH | 3013827,19 | 2698597,04 | 2078921,34 | 315230,14 | 619675,71 | 22,96 | 103,83 |

Table 10: **Group C**, Single Values per Stent Type and Vessel. Area within EEL, Area within IEL, Lumen, Media and Intima (µm^2^). Stenosis (%), Intimal Mean Thickness (µm).

| **Animal no.** | **Side** | **Artery** | **Stent type** | **Area Within EEL (µm^2^)  Measured** | **Area Within IEL (µm^2^)  Measured** | **Lumen Area (µm^2^)  Measured** | **Media Area (µm^2^) Calculated** | **Intima Area (µm^2^) Calculated** | **% Stenosis Calculated** | **Intimal Mean  Thickness (µm)** |
| --- | --- | --- | --- | --- | --- | --- | --- | --- | --- | --- |
| 1067 | Left | Iliac | C | 3472695,28 | 2990665,20 | 2462213,13 | 482030,08 | 528452,08 | 17,67 | 78,90 |
| 1067 | Right |  | C | 3858810,17 | 3314614,52 | 2717495,22 | 544195,65 | 597119,29 | 18,01 | 92,89 |
| 1081 | Left |  | C | 1005731,96 | 741570,16 | 411153,17 | 264161,80 | 330417,00 | 44,56 | 69,42 |
| 1081 | Right |  | C | 3148852,45 | 2756317,09 | 2222709,42 | 392535,36 | 533607,66 | 19,36 | 90,94 |
| 1091 | Left |  | C | 3758413,43 | 3297183,18 | 2887208,72 | 461230,26 | 409974,46 | 12,43 | 69,54 |
| 1091 | Right |  | C | 2904423,51 | 2597158,93 | 1830301,26 | 307264,58 | 766857,67 | 29,53 | 139,84 |

Table 11: **Group D**, Single Values per Stent Type and Vessel. Area within EEL, Area within IEL, Lumen, Media and Intima (µm^2^). Stenosis (%), Intimal Mean Thickness (µm).

| **Animal no.** | **Side** | **Artery** | **Stent type** | **Area Within EEL (µm^2^)  Measured** | **Area Within IEL (µm^2^)  Measured** | **Lumen Area (µm^2^)  Measured** | **Media Area (µm^2^) Calculated** | **Intima Area (µm^2^) Calculated** | **% Stenosis Calculated** | **Intimal Mean  Thickness (µm)** |
| --- | --- | --- | --- | --- | --- | --- | --- | --- | --- | --- |
| 1079 | Left | Iliac | D | 4408626,89 | 4096667,37 | 3285513,81 | 311959,52 | 811153,56 | 19,80 | 118,41 |
| 1079 | Right |  | D | 4598621,82 | 3990297,43 | 3711445,12 | 608324,39 | 278852,31 | 6,99 | 37,25 |
| 1082 | Left |  | D | 4158129,36 | 3711613,09 | 3188481,22 | 446516,27 | 523131,87 | 14,09 | 78,13 |
| 1082 | Right |  | D | 4388331,38 | 3868942,20 | 3291935,42 | 519389,18 | 577006,78 | 14,91 | 116,18 |
| 1092 | Left |  | D | 3200529,24 | 2933919,87 | 2425663,49 | 266609,37 | 508256,38 | 17,32 | 82,88 |
| 1092 | Right |  | D | 4292236,32 | 3811116,33 | 3387093,49 | 481119,99 | 424022,84 | 11,13 | 51,40 |

Table 12: **Group EF**, Single Values per Stent Type and Vessel. Area within EEL, Area within IEL, Lumen, Media and Intima (µm^2^). Stenosis (%), Intimal Mean Thickness (µm).

| **Animal no.** | **Side** | **Artery** | **Stent type** | **Area Within EEL (µm^2^)  Measured** | **Area Within IEL (µm^2^)  Measured** | **Lumen Area (µm^2^)  Measured** | **Media Area (µm^2^) Calculated** | **Intima Area (µm^2^) Calculated** | **% Stenosis Calculated** | **Intimal Mean  Thickness (µm)** |
| --- | --- | --- | --- | --- | --- | --- | --- | --- | --- | --- |
| 1070 | Left | Iliac | EF | 2798022,01 | 2338635,63 | 2009961,66 | 459386,38 | 328673,97 | 14,05 | 10,47 |
| 1070 | Right |  | EF | 1385470,73 | 875941,36 | 425123,94 | 509529,38 | 450817,42 | 51,47 | 39,68 |
| 1083 | Left |  | EF | 3852383,11 | 3234207,87 | 2736957,97 | 618175,24 | 497249,91 | 15,37 | 82,55 |
| 1083 | Right |  | EF | 1961931,79 | 1409029,47 | 992635,69 | 552902,33 | 416393,78 | 29,55 | 75,92 |
| 1093 | Left |  | EF | 3163548,76 | 2854181,94 | 2694725,05 | 309366,82 | 159456,89 | 5,59 | 24,33 |
| 1093 | Right |  | EF | 3152747,08 | 2910195,48 | 2685850,34 | 242551,61 | 224345,13 | 7,71 | 20,10 |

Table 13: Table 14: **Group EG**, Single Values per Stent Type and Vessel. Area within EEL, Area within IEL, Lumen, Media and Intima (µm^2^). Stenosis (%), Intimal Mean Thickness (µm).

| **Animal no.** | **Side** | **Artery** | **Stent type** | **Area Within EEL (µm^2^)  Measured** | **Area Within IEL (µm^2^)  Measured** | **Lumen Area (µm^2^)  Measured** | **Media Area (µm^2^) Calculated** | **Intima Area (µm^2^) Calculated** | **% Stenosis Calculated** | **Intimal Mean  Thickness (µm)** |
| --- | --- | --- | --- | --- | --- | --- | --- | --- | --- | --- |
| 1071 | Left | Iliac | EG | 3453101,25 | 3001899,74 | 2667700,28 | 451201,51 | 334199,46 | 11,13 | 41,58 |
| 1071 | Right |  | EG | 4305917,52 | 3565678,07 | 3202548,10 | 740239,45 | 363129,97 | 10,18 | 50,46 |
| 1084 | Left |  | EG | 2934178,43 | 2545074,61 | 2276765,92 | 389103,81 | 268308,69 | 10,54 | 28,66 |
| 1084 | Right |  | EG | 3059469,19 | 2635419,44 | 2450372,06 | 424049,75 | 185047,38 | 7,02 | 45,59 |
| 1094 | Left |  | EG | 3232928,17 | 2912919,99 | 2328822,19 | 320008,18 | 584097,80 | 20,05 | 81,71 |
| 1094 | Right |  | EG | 3380632,61 | 2898638,09 | 2653697,68 | 481994,52 | 244940,41 | 8,45 | 37,60 |

## Supplement III. Statistical data

Table 15: Descriptive Statistics from Medial Area calculation per Group (Ends and Middle segment combined) and Shapiro-Wilk normality test

| **Group** | **C** | **D** | **AL** | **AM** | **AH** | **BL** | **BM** | **BH** | **EF** | **EG** |
| --- | --- | --- | --- | --- | --- | --- | --- | --- | --- | --- |
| Number of values | 6 | 6 | 6 | 6 | 6 | 6 | 4 | 6 | 6 | 6 |
| Minimum | 264162 | 266609 | 226020 | 229720 | 221404 | 200675 | 233797 | 207045 | 242552 | 320008 |
| 25% Percentile | 296489 | 300622 | 278395 | 261781 | 309376 | 255148 | 260904 | 256452 | 292663 | 371830 |
| Median | 426883 | 463818 | 322439 | 301294 | 443875 | 323482 | 354500 | 311488 | 484458 | 437626 |
| 75% Percentile | 497571 | 541623 | 372676 | 404092 | 599480 | 420091 | 433114 | 498952 | 569221 | 546556 |
| Maximum | 544196 | 608324 | 398786 | 428923 | 789535 | 512366 | 455228 | 642780 | 618175 | 740239 |
| Range | 280034 | 341715 | 172766 | 199203 | 568131 | 311691 | 221431 | 435735 | 375624 | 420231 |
| Mean | 408570 | 438986 | 321585 | 321586 | 462253 | 337107 | 349506 | 366122 | 448652 | 467766 |
| Std. Deviation | 107645 | 128702 | 59518 | 76326 | 194811 | 110259 | 91135 | 157312 | 145124 | 144672 |
| Std. Error of Mean | 43946 | 52543 | 24298 | 31160 | 79531 | 45013 | 45568 | 64222 | 59247 | 59062 |
| Lower 95% CI of mean | 295603 | 303922 | 259124 | 241487 | 257811 | 221397 | 204490 | 201033 | 296353 | 315942 |
| Upper 95% CI of mean | 521536 | 574051 | 384046 | 401685 | 666695 | 452817 | 494522 | 531211 | 600951 | 619590 |
| Sum | 2451418 | 2633919 | 1929510 | 1929514 | 2773518 | 2022643 | 1398025 | 2196732 | 2691912 | 2806597 |
| **Shapiro-Wilk normality test** | | | | | | | | | | |
| W | 0,9546 | 0,9534 | 0,9805 | 0,9377 | 0,9645 | 0,9567 | 0,9800 | 0,8845 | 0,9381 | 0,8524 |
| P value | 0,7773 | 0,7673 | 0,9538 | 0,6408 | 0,8538 | 0,7940 | 0,9020 | 0,2902 | 0,6439 | 0,1646 |
| Passed normality test (alpha=0.05)? | Yes | Yes | Yes | Yes | Yes | Yes | Yes | Yes | Yes | Yes |

Table 16: Descriptive Statistics from Intimal Area calculation per Group (Ends and Middle segment combined) and Shapiro-Wilk normality test

| **Group** | **C** | **D** | **AL** | **AM** | **AH** | **BL** | **BM** | **BH** | **EF** | **EG** |
| --- | --- | --- | --- | --- | --- | --- | --- | --- | --- | --- |
| Number of values | 6 | 6 | 6 | 6 | 6 | 6 | 4 | 6 | 6 | 6 |
| Minimum | 330417 | 278852 | 221466 | 337784 | 450750 | 304307 | 333857 | 257711 | 159457 | 185047 |
| 25% Percentile | 390085 | 387730 | 302036 | 362371 | 465985 | 356719 | 362708 | 352177 | 208123 | 229967 |
| Median | 531030 | 515694 | 391413 | 506227 | 672435 | 418025 | 557458 | 577903 | 372534 | 301254 |
| 75% Percentile | 639554 | 635543 | 707774 | 710281 | 769252 | 552984 | 680065 | 625135 | 462426 | 418372 |
| Maximum | 766858 | 811154 | 821976 | 940954 | 797239 | 584363 | 684868 | 641514 | 497250 | 584098 |
| Range | 436441 | 532301 | 600510 | 603171 | 346489 | 280056 | 351011 | 383803 | 337793 | 399050 |
| Mean | 527738 | 520404 | 470811 | 549191 | 637308 | 440239 | 533410 | 509729 | 346156 | 329954 |
| Std. Deviation | 151546 | 176369 | 227702 | 221504 | 149940 | 104967 | 170617 | 153734 | 133204 | 139809 |
| Std. Error of Mean | 61868 | 72002 | 92959 | 90429 | 61213 | 42852 | 85309 | 62761 | 54380 | 57077 |
| Lower 95% CI of mean | 368700 | 335316 | 231852 | 316737 | 479955 | 330083 | 261920 | 348395 | 206367 | 183233 |
| Upper 95% CI of mean | 686776 | 705492 | 709770 | 781646 | 794660 | 550395 | 804900 | 671062 | 485945 | 476675 |
| Sum | 3166428 | 3122424 | 2824868 | 3295149 | 3823845 | 2641434 | 2133640 | 3058372 | 2076937 | 1979724 |
| **Shapiro-Wilk normality test** | | | | | | | | | | |
| W | 0,9692 | 0,9605 | 0,9131 | 0,9002 | 0,8784 | 0,9436 | 0,8801 | 0,8317 | 0,9382 | 0,8939 |
| P value | 0,8870 | 0,8238 | 0,4569 | 0,3752 | 0,2617 | 0,6880 | 0,3389 | 0,1110 | 0,6449 | 0,3390 |
| Passed normality test (alpha=0.05)? | Yes | Yes | Yes | Yes | Yes | Yes | Yes | Yes | Yes | Yes |

Table 17: Descriptive Statistics from Stenosis calculation per Group (Ends and Middle segment combined) and Shapiro-Wilk normality test

| **Group** | **C** | **D** | **AL** | **AM** | **AH** | **BL** | **BM** | **BH** | **EF** | **EG** |
| --- | --- | --- | --- | --- | --- | --- | --- | --- | --- | --- |
| Number of values | 6 | 6 | 6 | 6 | 6 | 6 | 4 | 6 | 6 | 6 |
| Minimum | 12,43 | 6,990 | 10,72 | 12,59 | 15,57 | 13,15 | 9,110 | 11,11 | 5,590 | 7,020 |
| 25% Percentile | 16,36 | 10,10 | 11,22 | 12,68 | 17,39 | 13,30 | 10,33 | 14,46 | 7,180 | 8,093 |
| Median | 18,69 | 14,50 | 14,92 | 19,30 | 25,21 | 15,16 | 19,13 | 17,82 | 14,71 | 10,36 |
| 75% Percentile | 33,29 | 17,94 | 36,15 | 30,44 | 35,44 | 18,57 | 35,81 | 21,13 | 35,03 | 13,36 |
| Maximum | 44,56 | 19,80 | 37,67 | 41,51 | 39,17 | 20,96 | 39,66 | 22,96 | 51,47 | 20,05 |
| Range | 32,13 | 12,81 | 26,95 | 28,92 | 23,60 | 7,810 | 30,55 | 11,85 | 45,88 | 13,03 |
| Mean | 23,59 | 14,04 | 20,88 | 22,03 | 26,22 | 15,92 | 21,76 | 17,63 | 20,62 | 11,23 |
| Std. Deviation | 11,69 | 4,536 | 12,37 | 11,00 | 9,386 | 3,045 | 13,50 | 4,089 | 17,29 | 4,579 |
| Std. Error of Mean | 4,774 | 1,852 | 5,049 | 4,490 | 3,832 | 1,243 | 6,751 | 1,669 | 7,059 | 1,869 |
| Lower 95% CI of mean | 11,32 | 9,280 | 7,895 | 10,48 | 16,37 | 12,73 | 0,2730 | 13,34 | 2,478 | 6,423 |
| Upper 95% CI of mean | 35,86 | 18,80 | 33,85 | 33,57 | 36,07 | 19,12 | 43,24 | 21,92 | 38,77 | 16,03 |
| Sum | 141,6 | 84,24 | 125,3 | 132,2 | 157,3 | 95,54 | 87,03 | 105,8 | 123,7 | 67,37 |
| **Shapiro-Wilk normality test** | | | | | | | | | | |
| W | 0,8501 | 0,9812 | 0,7678 | 0,8724 | 0,9411 | 0,8952 | 0,9424 | 0,9723 | 0,8549 | 0,8007 |
| P value | 0,1576 | 0,9573 | 0,0296 | 0,2360 | 0,6678 | 0,3466 | 0,6691 | 0,9074 | 0,1723 | 0,0597 |
| Passed normality test (alpha=0.05)? | Yes | Yes | No | Yes | Yes | Yes | Yes | Yes | Yes | Yes |

Table 18: Descriptive Statistics from Intimal Thickness calculation per Group (Ends and Middle segment combined) and Shapiro-Wilk normality test

| **Group** | **C** | **D** | **AL** | **AM** | **AH** | **BL** | **BM** | **BH** | **EF** | **EG** |
| --- | --- | --- | --- | --- | --- | --- | --- | --- | --- | --- |
| Number of values | 6 | 6 | 6 | 6 | 6 | 6 | 4 | 6 | 6 | 6 |
| Minimum | 69,42 | 37,25 | 46,15 | 36,77 | 92,66 | 54,89 | 47,38 | 37,24 | 10,47 | 28,66 |
| 25% Percentile | 69,51 | 47,86 | 46,49 | 59,58 | 98,23 | 63,40 | 51,89 | 51,60 | 17,69 | 35,37 |
| Median | 84,92 | 80,51 | 52,85 | 77,89 | 107,4 | 71,64 | 88,64 | 79,08 | 32,01 | 43,59 |
| 75% Percentile | 104,6 | 116,7 | 109,2 | 108,3 | 111,0 | 88,71 | 130,6 | 98,28 | 77,58 | 58,27 |
| Maximum | 139,8 | 118,4 | 127,0 | 143,2 | 112,5 | 107,2 | 136,8 | 103,8 | 82,55 | 81,71 |
| Range | 70,42 | 81,16 | 80,89 | 106,4 | 19,79 | 52,34 | 89,46 | 66,59 | 72,08 | 53,05 |
| Mean | 90,26 | 80,71 | 71,46 | 83,26 | 105,1 | 75,69 | 90,38 | 75,34 | 42,18 | 47,60 |
| Std. Deviation | 26,30 | 32,98 | 34,81 | 35,38 | 7,498 | 17,87 | 41,20 | 26,25 | 30,29 | 18,28 |
| Std. Error of Mean | 10,74 | 13,46 | 14,21 | 14,44 | 3,061 | 7,297 | 20,60 | 10,72 | 12,36 | 7,463 |
| Lower 95% CI of mean | 62,66 | 46,10 | 34,93 | 46,13 | 97,24 | 56,94 | 24,82 | 47,79 | 10,39 | 28,42 |
| Upper 95% CI of mean | 117,9 | 115,3 | 108,0 | 120,4 | 113,0 | 94,45 | 155,9 | 102,9 | 73,96 | 66,78 |
| Sum | 541,5 | 484,3 | 428,7 | 499,6 | 630,6 | 454,2 | 361,5 | 452,0 | 253,1 | 285,6 |
| **Shapiro-Wilk normality test** | | | | | | | | | | |
| W | 0,8091 | 0,9213 | 0,7688 | 0,9495 | 0,9086 | 0,9209 | 0,9406 | 0,9230 | 0,8756 | 0,8677 |
| P value | 0,0708 | 0,5151 | 0,0303 | 0,7362 | 0,4275 | 0,5116 | 0,6582 | 0,5275 | 0,2496 | 0,2171 |
| Passed normality test (alpha=0.05)? | Yes | Yes | No | Yes | Yes | Yes | Yes | Yes | Yes | Yes |

## Supplement IV. Morphometry images

Figure 1: Animal No. 1081, Right Iliac artery, Group C.


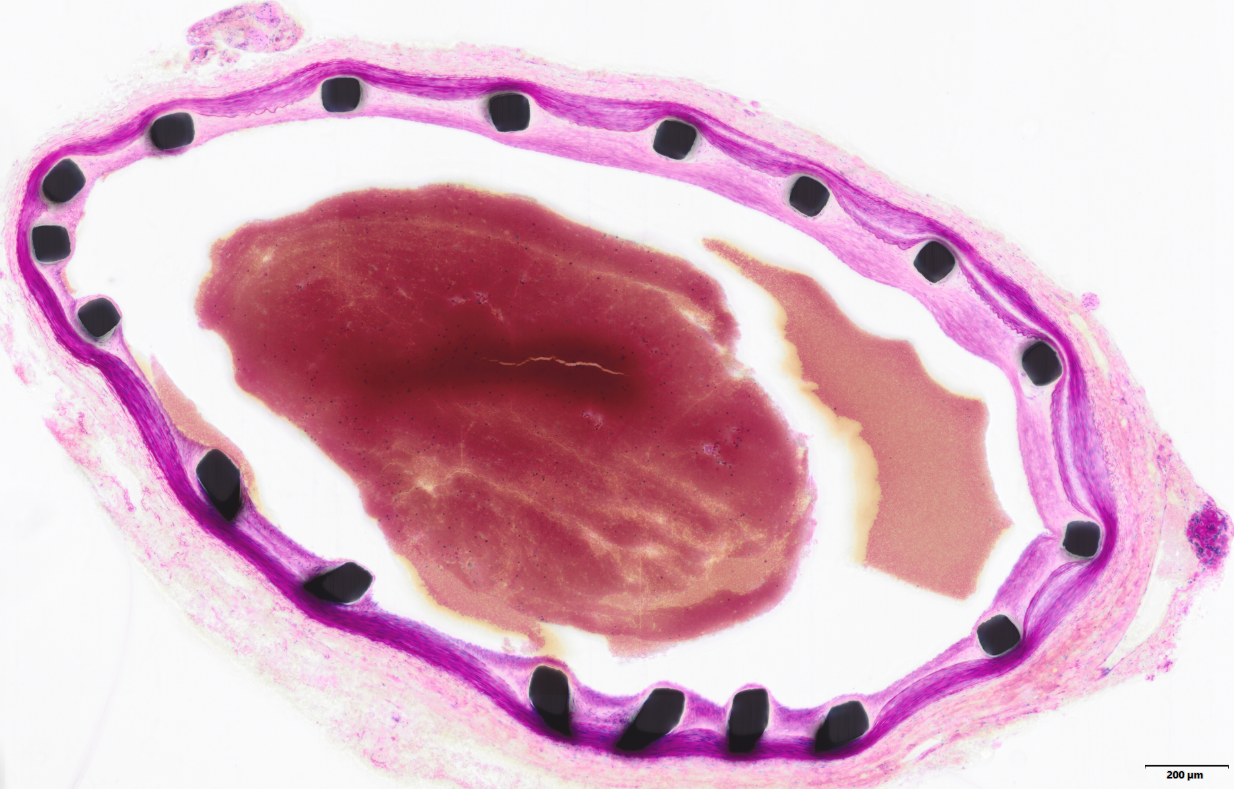


Figure 2: Animal No. 1082, Left Iliac Artery, Group D.


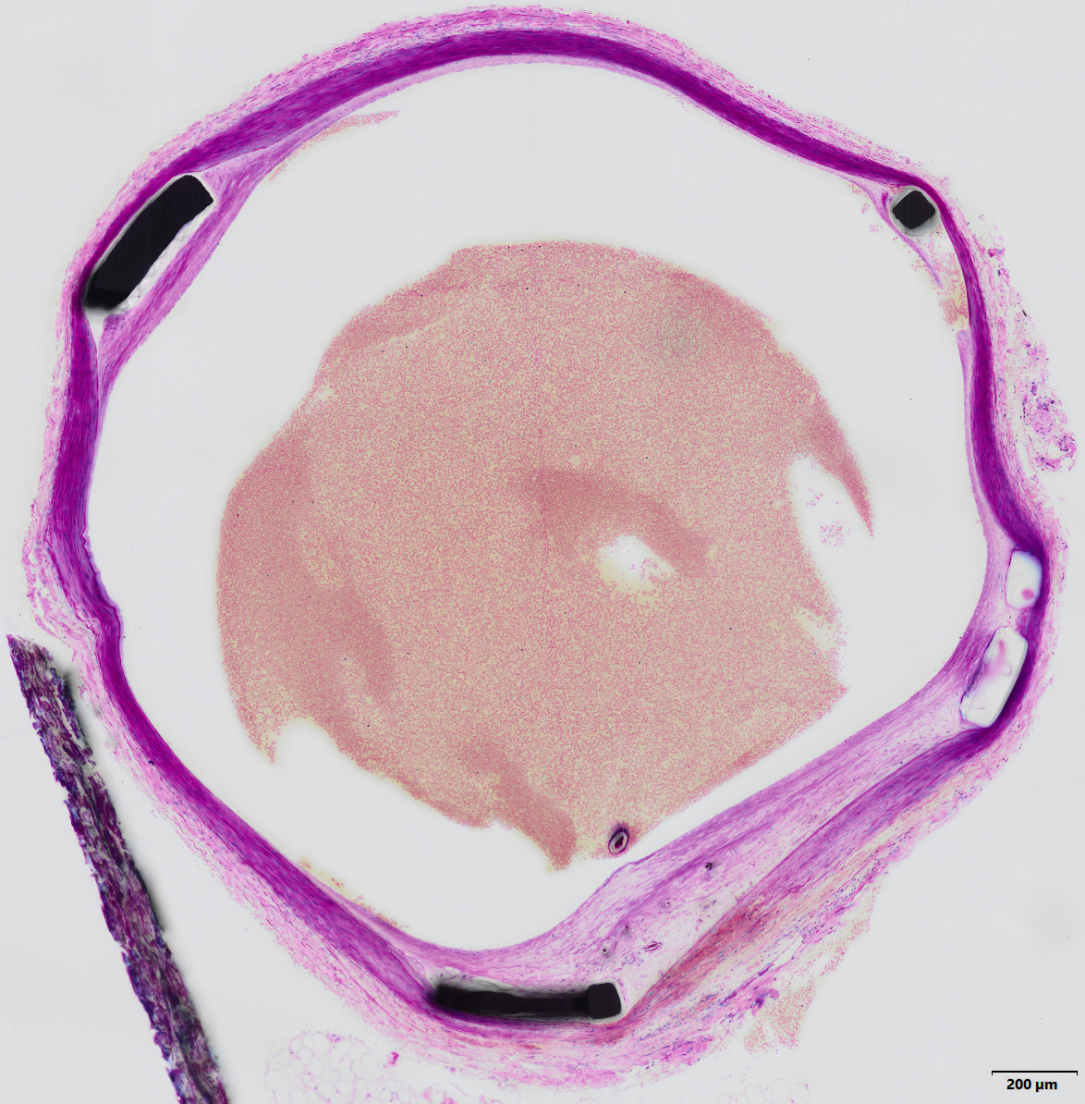


Figure 3: Animal No. 1085, Left Iliac Artery, Group AL.


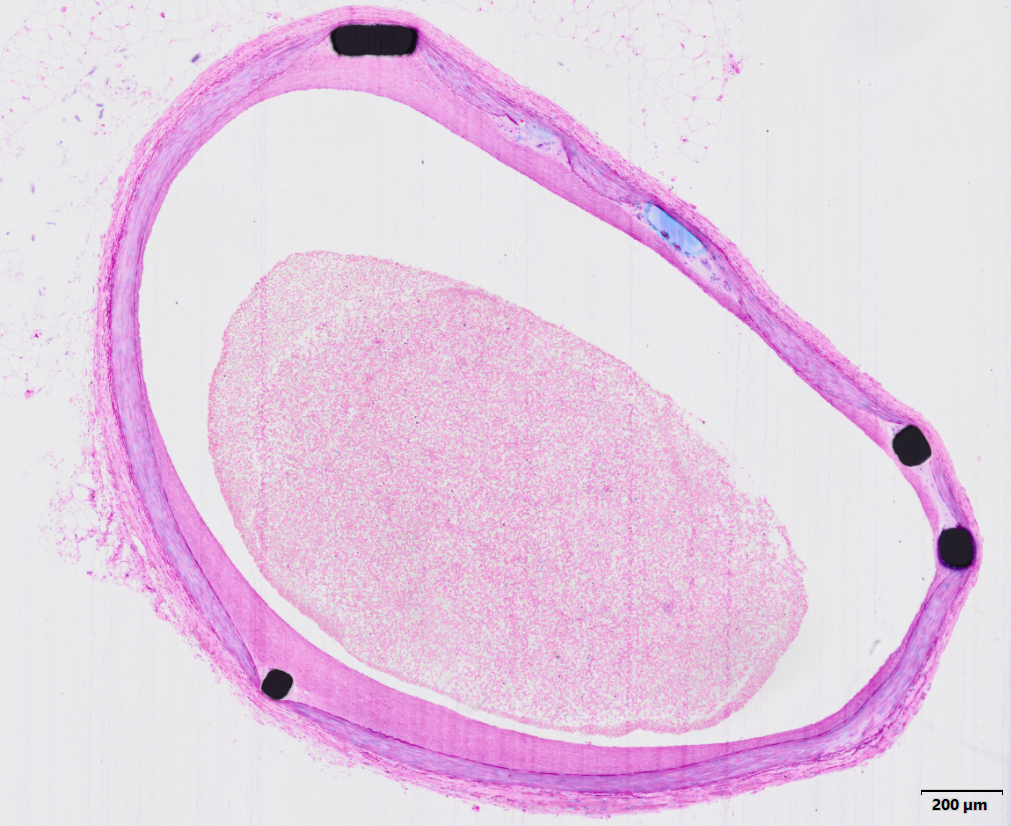


Figure 4: Animal No. 1086, Left Iliac Artery, Group AM.


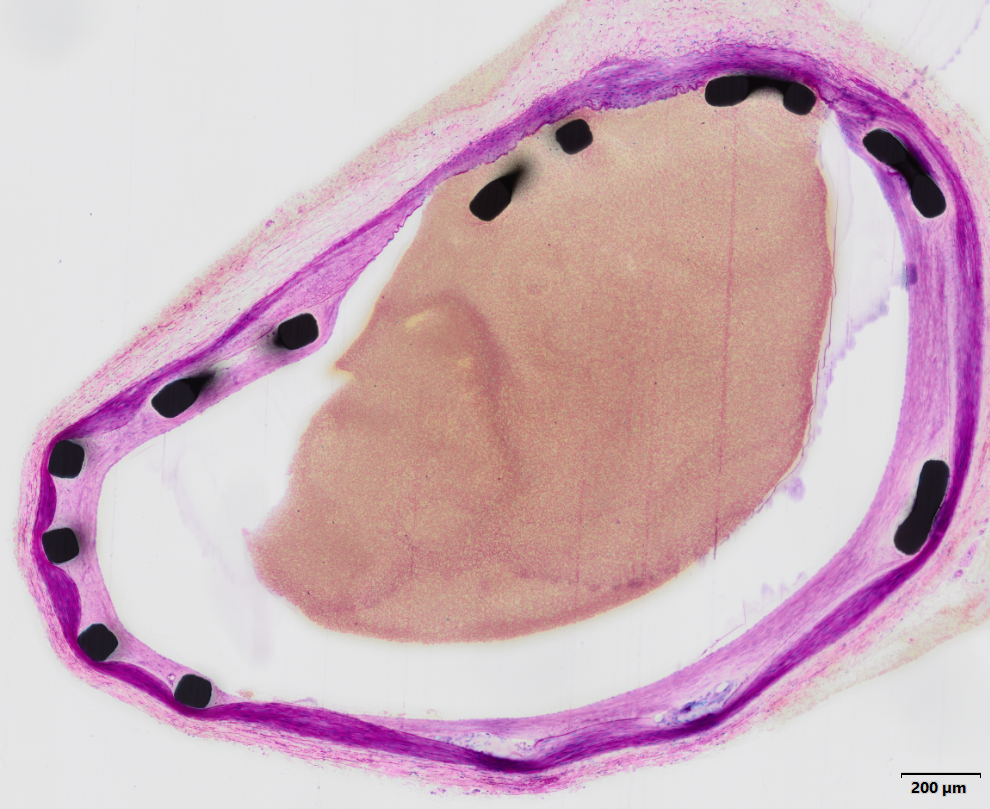


Figure 5: Animal No. 1087, Right Iliac Artery, Group AH.


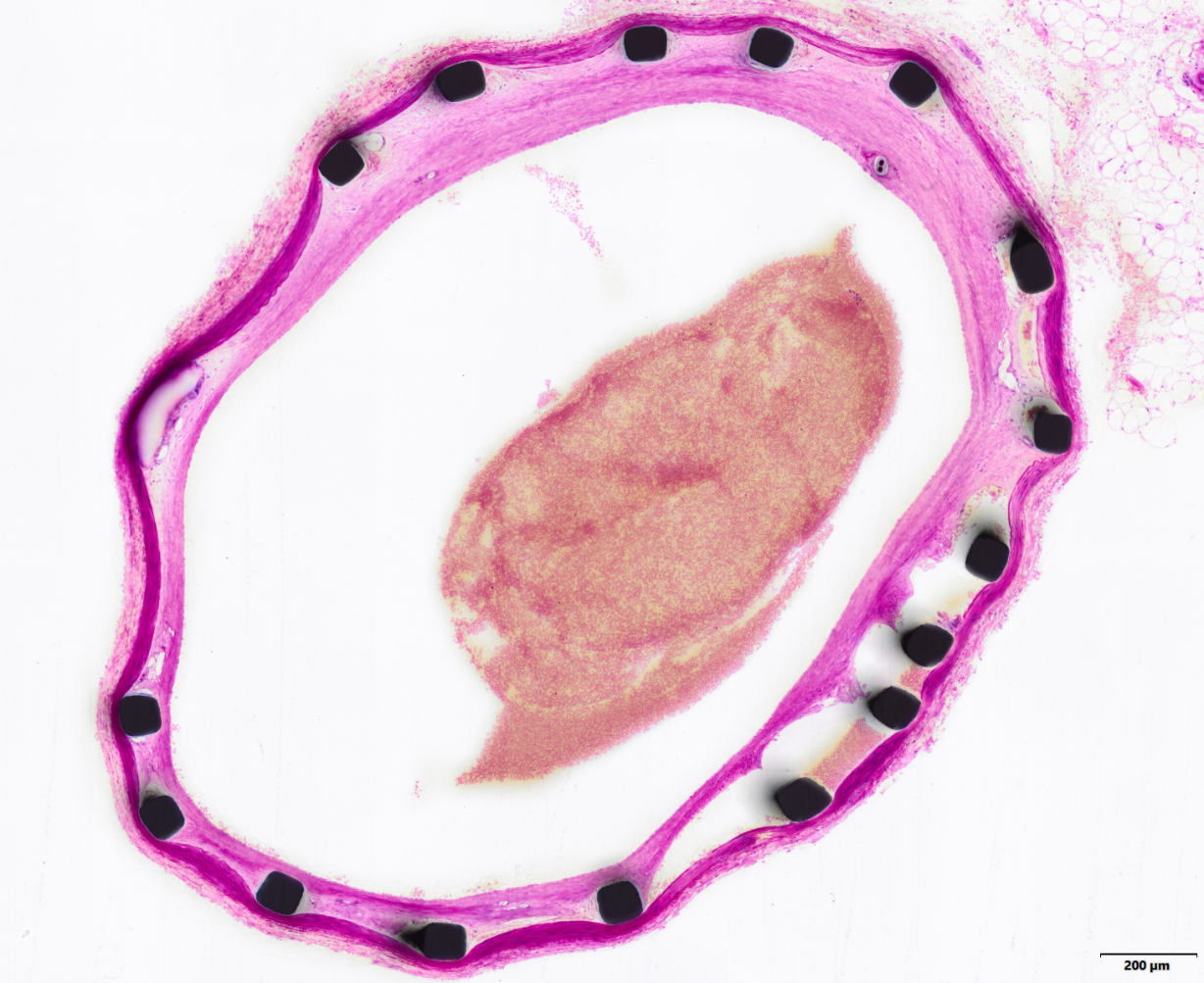


Figure 6: Animal No. 1075, Right Iliac Artery, Group BL.


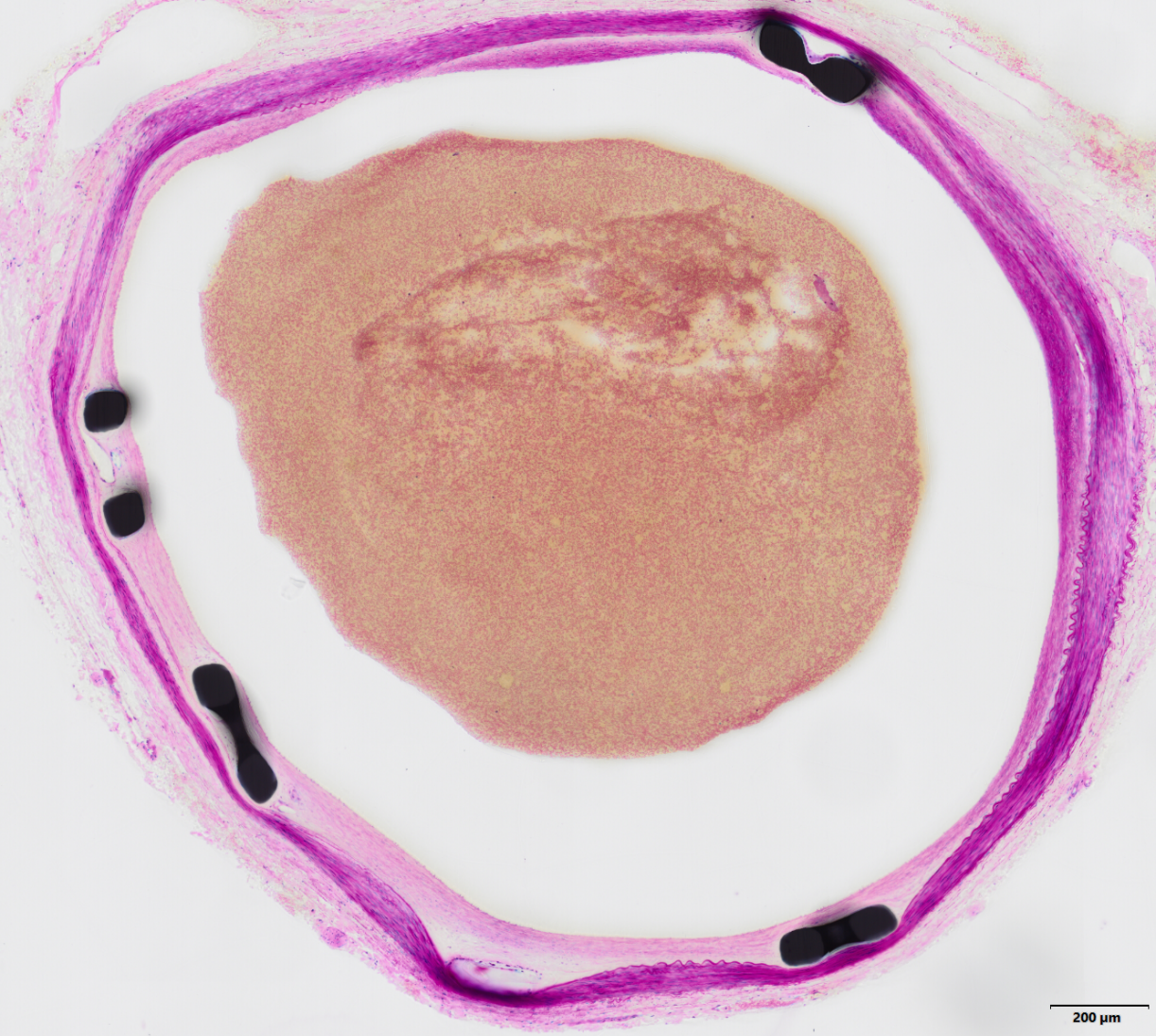


Figure 7: Animal No. 1064, Right Iliac Artery, Group BM.


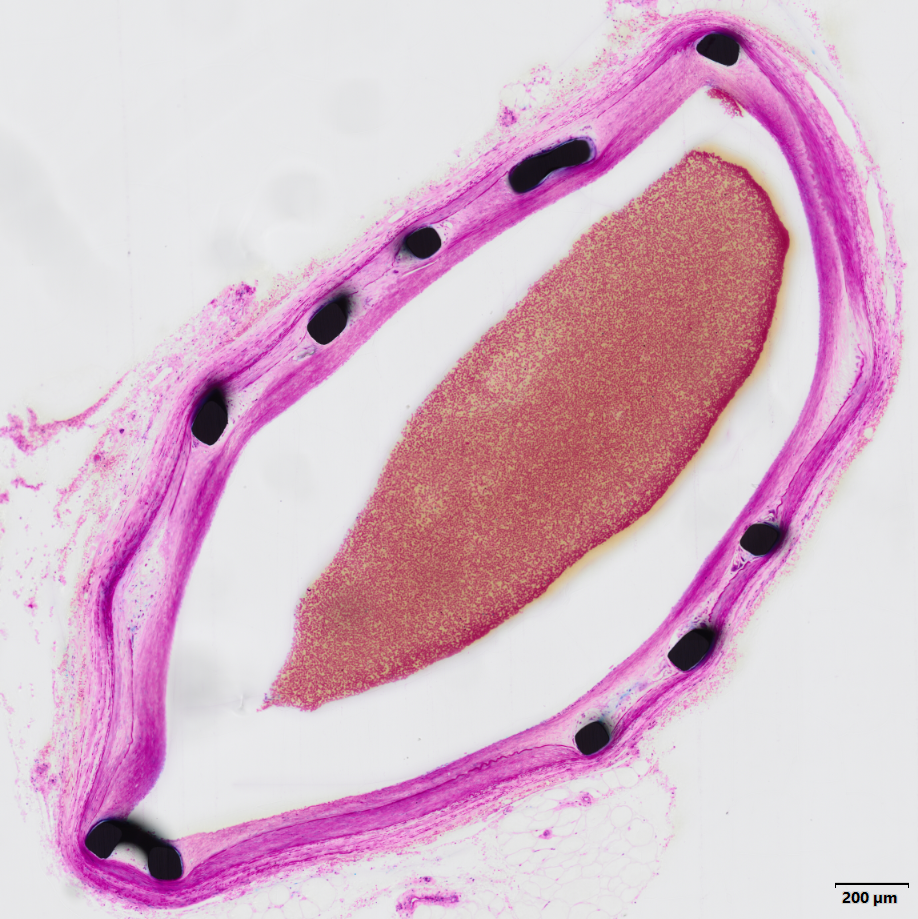


Figure 8: Animal No. 1069, Right Iliac Artery, Group BH.


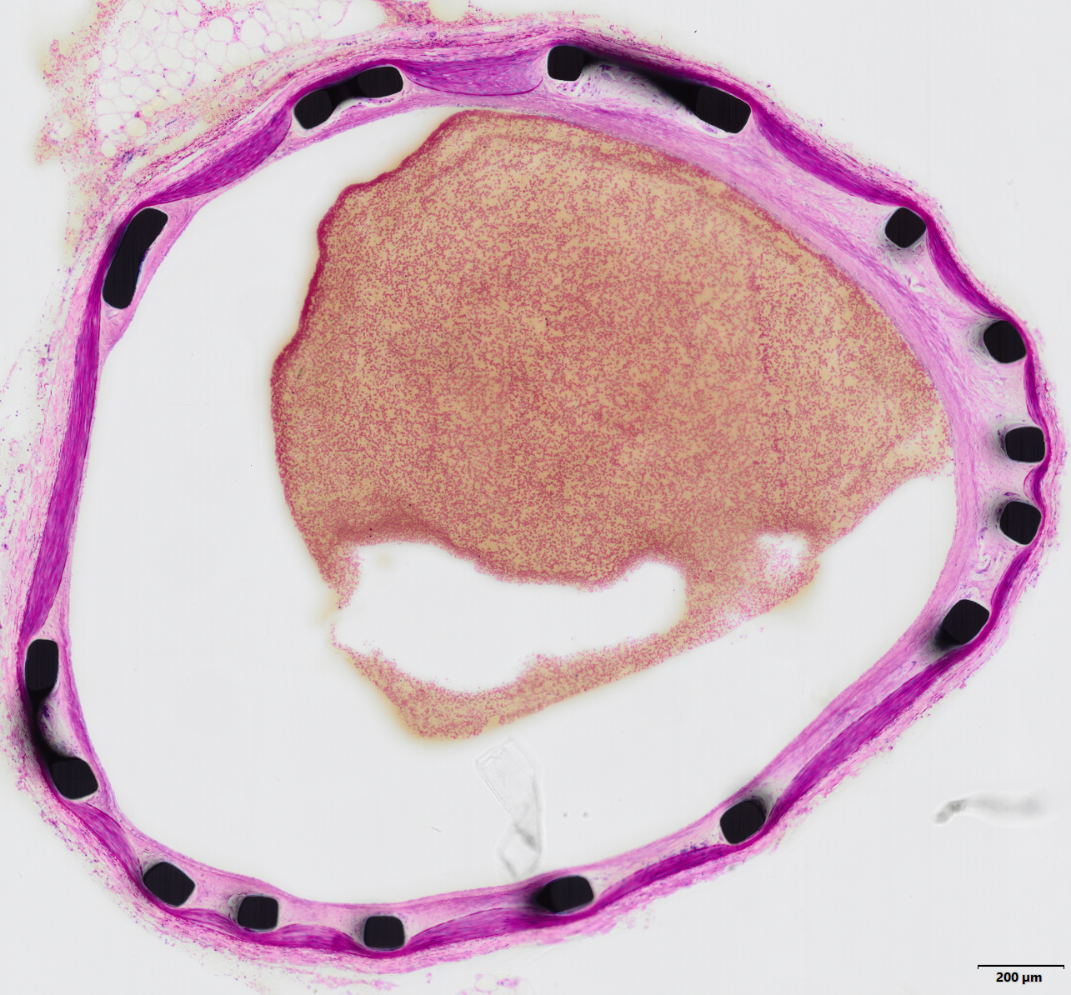


Figure 9: Animal No. 1083, Right Iliac Artery, Group EF.


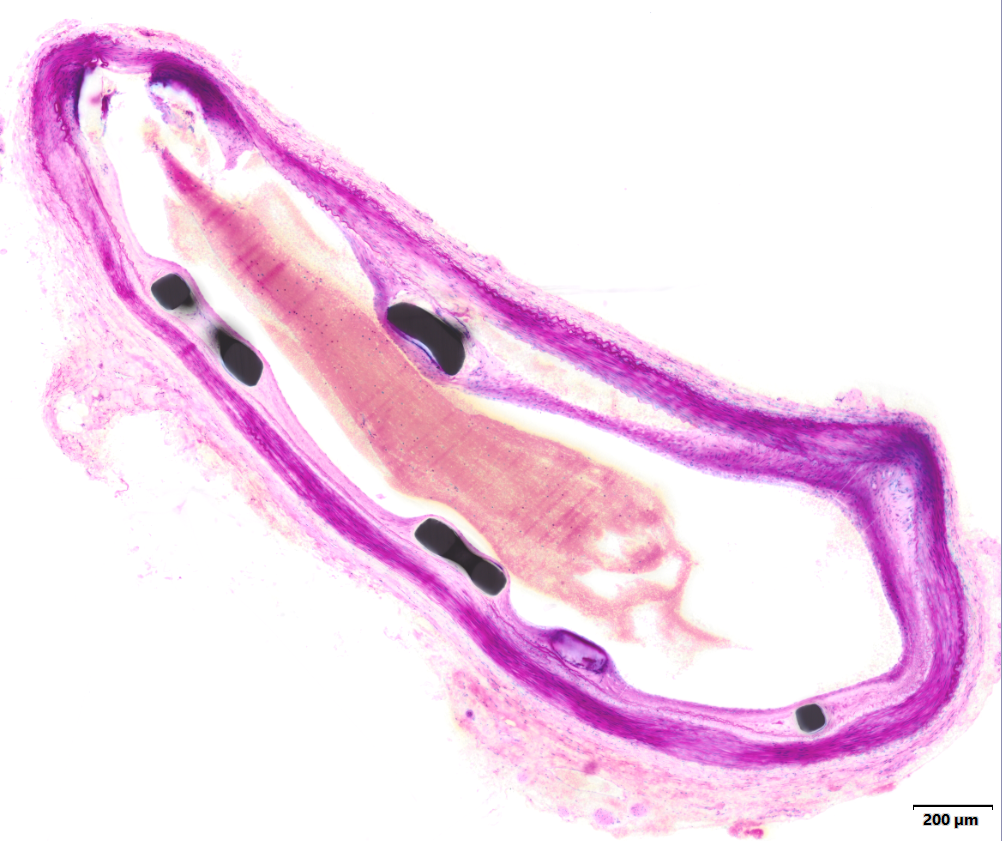


Figure 10: Animal No. 1071, Left Iliac Artery, Group EG.


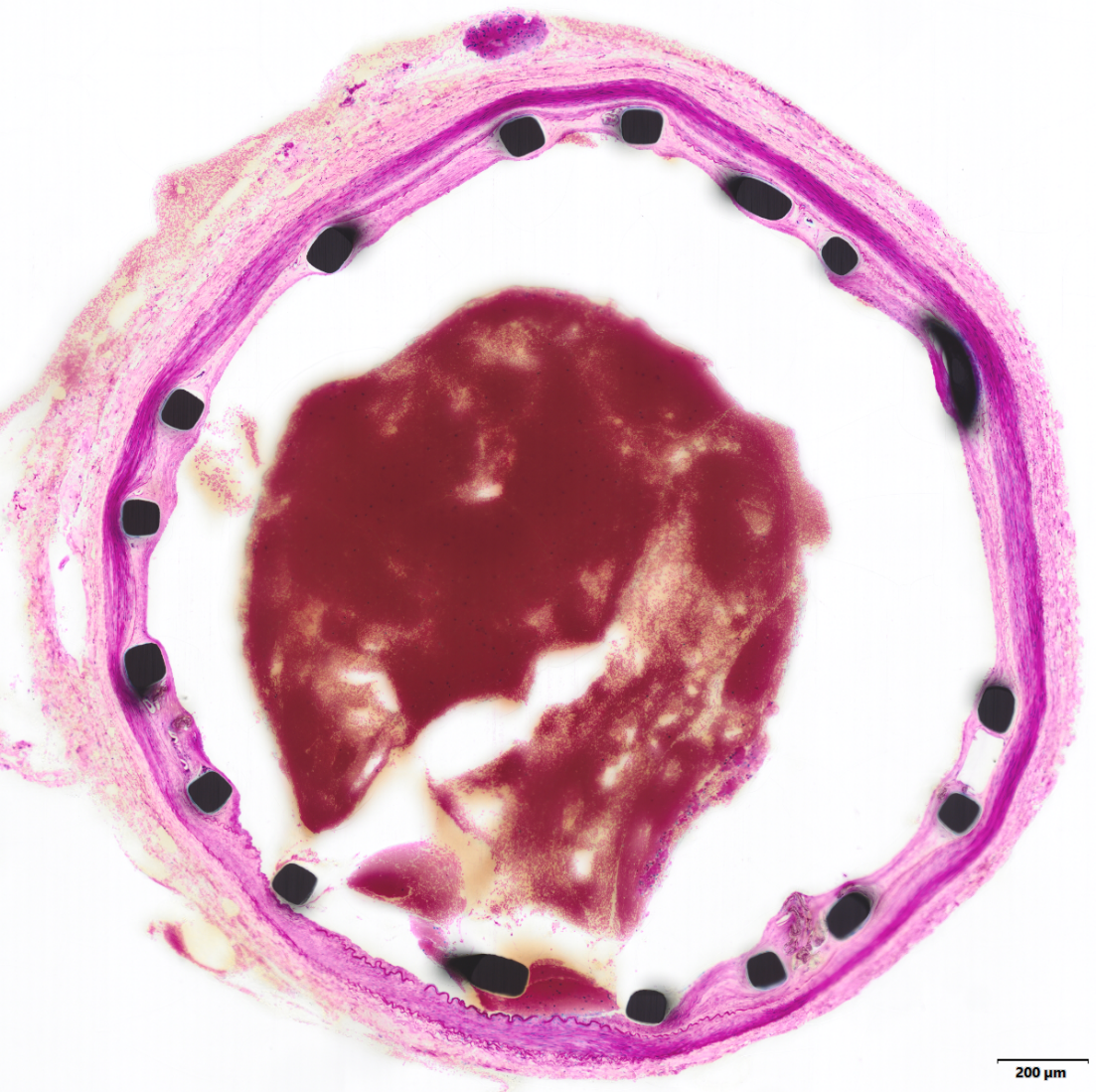

Supplement: Supplementary file 1 — Supplementary Information. [file 41598_2022_16025_MOESM1_ESM.docx]
